# Educational utility of observational workplace-based assessment modalities in Australian vocational general practice training: a cross-sectional study

**DOI:** 10.1186/s12909-025-07328-y

**Published:** 2025-05-23

**Authors:** Alison Fielding, Benjamin Mundy, Amanda Tapley, Sarah Gani, Rula Ali, Michael Bentley, Rachael Boland, Lina Zbaidi, Elizabeth Holliday, Jean Ball, Mieke van Driel, Linda Klein, Parker Magin

**Affiliations:** 1 School of Medicine & Public Health, University of Newcastle, University Drive, Callaghan, NSW 2308 Australia; 2 General Practice Training Research, Royal Australian College of General Practitioners, Level 1, 20 Mclntosh Drive, Mayfield West, NSW 2304 Australia; 3 General Practice Training Medical Education, Royal Australian College of General Practitioners, Suite 2, 16 Napier Close, Deakin, ACT 2600 Australia; 4 Discipline of General Practice, Faculty of Medicine and Health, University of New South Wales, Sydney, NSW 2052 Australia; 5 General Practice Training Research, Royal Australian College of General Practitioners, TAS, 62 Patrick Street, Hobart, 7000 Australia; 6 General Practice Training Medical Education, GP Training, Royal Australian College of General Practitioners, TAS, 62 Patrick Street, Hobart, 7000 Australia; 7 Northern Territory General Practice Education, Level 3, Building 1, Yellow Precinct, Charles Darwin University, Ellengowan Drive, Casuarina, NT 0810 Australia; 8 Clinical Research Design IT and Statistical Support Unit (CReDITSS), Hunter Medical Research Institute (HMRI), 1 Kookaburra Circuit, New Lambton Heights, NSW 2305 Australia; 9 General Practice Clinical Unit, Faculty of Medicine, Mayne Medical Building, The University of Queensland, Level 2288 Herston Road, Herston, QLD 4006 Australia

**Keywords:** Education, medical, Education, Medical, Graduate, Educational Measurement (or Assessment, Educational), General Practice, Family Practice, Formative Feedback

## Abstract

**Background:**

Direct observation, workplace-based assessments (WBAs) are a fundamental component of competency-based postgraduate medical education. In Australian general practice vocational training, external clinical teaching visits (ECTVs) are key observation-based WBAs. Traditionally, ECTVs are conducted face-to-face, but the COVID-19 pandemic saw the development and implementation of remote ECTV modalities. It remains unknown if perceived educational utility of remote ECTVs differs from traditional face-to-face ECTVs. This study explored the educational utility of ECTVs, including face-to-face and remote formats.

**Methods:**

General practice trainees (‘registrars’) and external clinical teaching visitors (‘ECT visitors', who are independent experienced GP observers) each completed a cross-sectional questionnaire following individual ECTVs undertaken in 2020. Outcomes included overall educational utility of the ECTV as perceived by registrars, registrar ratings of likelihood to change their clinical practice as a result of the ECTV, registrar ratings of likelihood to change their approach to learning/training as a result of the ECTV, and overall educational utility of the ECTV as perceived by the ECT visitor. Educational utility ratings (5-point scales) were analysed descriptively. Univariable and multivariable logistic regression were employed to examine factors associated with dichotomised educational utility ratings.

**Results:**

Response rates were 41% (*n* = 801) for registrars and 39% (*n* = 742) for ECT visitors. Most registrars (64.1%) rated ECTV overall educational utility as ‘very useful’; 58.5% and 47.9% of registrars rated their likelihood to change practice and approach to learning/training, respectively, as ‘very likely’. No statistically significant differences in perceived educational utility ratings were identified between face-to-face and remote video/phone ECTVs (multivariable p-value range: .07-.96). Receiving feedback that was focused/specific/easy to translate into action was consistently associated with registrars’ rating overall educational utility as ‘very useful’ (odds ratio (OR): 12.8, 95% confidence interval (CI): 8.26 to19.9), rating likelihood to change practice as ‘very likely’ (OR: 2.5, 95%CI: 1.59 to 3.94), and rating likelihood to change learning/training approach as ‘very likely’ (OR: 3.19, 95%CI: 1.97 to 5.17).

**Conclusions:**

ECTVs are perceived by registrars and ECT visitors to be educationally useful across different delivery modalities and formats. The quality and features of the feedback provided appear most important in ECTVs as an assessment for learning.

**Supplementary Information:**

The online version contains supplementary material available at 10.1186/s12909-025-07328-y.

## Background

Workplace-based assessment (WBA) is a central feature of the assessment of general practitioner (GP) trainees (‘registrars’) as they develop competency in general practice [1]. Direct observation-based assessments in the workplace are recognised as a fundamental component of competency-based medical education [2]. Direct observation WBAs are implemented in general practice/family medicine training programs in Australia, and in other countries that share similarities with Australia, including Canada [3], New Zealand [4], Ireland [5], and the Netherlands [6, 7].

In Australian vocational general practice training, external clinical teaching visits (ECTVs) are used extensively as a direct observation, workplace-based assessment. ECTVs involve an independent, experienced GP (the ‘ECT visitor’) observing the GP registrar during a clinical session (approximately three hours in duration) [8]. The ECT visitor, who is external to the registrar’s practice, provides structured feedback regarding the registrar’s observed strengths and weaknesses, intending for this feedback to be translated into the registrar’s practice for ongoing competency development [9]. ECTVs provide a vital formative function in Australia’s apprenticeship-style vocational GP training model, where registrars practice with substantial autonomy. [10].

Within a theory-based framework for programmatic assessment [11], ECTVs are an example of a WBA that serve to function as both a low-stakes assessment *of* learning and an assessment *for* learning. Specifically, while each ECTV contributes a low stakes data point for monitoring satisfactory competency development across the course of training, each single ECTV also provides a potentially rich opportunity *for* learning [11].

Although ECTVs are anecdotally highly regarded, empirical evidence of their educational utility as a formative assessment is limited. Previous research has typically examined specific forms of WBAs that can be conducted within an ECTV, for example, Mini-Clinical Evaluation Exercises [12–15], feedback forms [9], and Random Case Analysis (RCA) [8, 16]. One qualitative study [17] and unpublished internal evaluations indicate that ECTVs are highly educationally valued. However, to our knowledge there have been no published quantitative studies broadly assessing the perceived educational utility of ECTVs.

Traditionally, ECTVs are conducted face-to-face. Remote ECTV options (such as video-taped consultations) have sometimes been used when distance poses a significant barrier to in-person observation. Prior to the onset of the COVID-19 pandemic, uptake of remote ECTV modalities had been slow. This is despite the resource-intensive nature of face-to-face ECTVs. The COVID-19 pandemic resulted in major disruption to medical education worldwide. Delivery of educational elements, including assessments, were rapidly adapted to remote formats [18]. Within Australian vocational general practice training, remote ECTV modalities were developed and implemented. The rapid commencement of remote ECTVs was necessity-driven, largely preceding understanding of, and evidence for, their educational utility. It remains unknown if perceived educational utility of remote ECTVs differs from traditional face-to-face ECTVs.

Within traditional ECTVs, the context of the clinical environment (e.g., patient case-mix), the educational content (e.g., specific topics discussed) and the complex dynamics of the assessor-trainee relationship, all have the potential to impact the educational substrate and subsequent utility as an assessment *for* learning. This is true for most, if not all, assessments at the ‘does’ level of Miller’s pyramid [19], as emphasised by van de Vleuten et al. who note “the real world is non-standardised and haphazard” [11]. It is unclear how the interplay of contextual factors may relate to perceived educational utility within both face-to-face and remote ECTV formats. The need to investigate a broad range of variables that could influence the formative benefit of feedback arising from observed consultations in practice has been recognised as an important research endeavour in further informing assessment feedback process models [20].

Despite the partial return of face-to-face ECTVs since the onset of the COVID-19 pandemic, there remains interest in maintaining remote ECTV formats given their relative resource efficiency compared with face-to-face ECTVs. Obtaining a better understanding of the perceived educational utility of ECTVs, and formats/elements of ECTVs relating to perceived educational utility, is paramount for informing a best-practice approach to ECTV delivery.

This study initially intended to explore the educational utility of traditional face-to-face ECTVs. However, with the rapid pandemic-mandated implementation of remote ECTVs coinciding with the beginning of this study, our original aims were adapted accordingly [21]. The specific aims of the study as delivered during the pandemic were to:

1. establish the perceived educational utility of different ECTV modalities, from the perspective of registrars and ECT visitors; and
2. identify factors associated with perceived educational utility ratings of ECTVs.

## Methods

### Theoretical approach

General practice training comes from a context of pragmatism and tradition, which has been largely atheoretical [1]. However, there has been a shift to a programmatic assessment framework, which draws on the theoretical concept of assessment *for* learning [11]. We used this approach as empirical observations arising from this study have the potential to contribute to understanding of assessment *for* learning through the lens of a general practice, medical education, direct-observation WBA.

### Design and setting

This was an observational cross-sectional study. Data was collected prospectively using online questionnaires completed soon after each ECTV. Ethics approval for the study was granted by the University of Newcastle’s Human Research Ethics Committee (reference: H-2020–0037-02). Informed consent was obtained from all participants, indicated via voluntary completion of the questionnaire.

A comprehensive study methodology is available in the published protocol paper [21]. Briefly, the study included registrars and ECT visitors from three general practice regional training organisations (RTOs) – GP Synergy (New South Wales and the Australian Capital Territory), Northern Territory General Practice Education (NTGPE) and General Practice Training Tasmania (GPTT). At the time of the study, RTOs were region-specific, not-for-profit organisations that co-ordinated in-practice training and delivered out-of-practice educational programs/workshops to registrars and supervisors. GP Synergy, NTGPE and GPTT were responsible for delivering GP training across two Australian states and two territories, collectively training 38% of Australian GP registrars [22].

All registrars in general practice terms, and ECT visitors, who completed an ECTV within the study period (March 2020-January 2021) were invited to participate after each of their ECTVs via email invitation to complete an online questionnaire.

The ECTV modalities employed by participating RTOs during the study period were face-to-face ECTVs, live video/telephone ECTVs, Clinical Notes Analysis (CNA)-ECTV, and Case-Based Discussion (CBD-ECTV) (Table 1).

**Table 1:** Modalities of external clinical teaching visits (ECTVs)

ECTV modality	Description
Face-to-face ECTVs	The external clinical teaching (ECT) visitor observes the registrar in-person conducting patient consultations for one clinical session (approximately three hours duration)
Live video/telephone ECTVs	A registrar’s consultations are observed/heard in real time via videoconference or telephone by the ECT visitor
Clinical Notes Analysis (CNA)-ECTV	Using a standardised selection procedure, a registrar obtains medical records from eight recent cases. During a call via videoconference or telephone, the ECT visitor selects at least four to discuss with the registrar. In this modality, registrars are provided with an opportunity to reflect on, and provide rationales for, their clinical decisions. Similar to random case analysis, noting that selection of cases, while standardised, is not via random selection. Implemented as a standalone pandemic-mandated replacement for face-to-face or live video/telephone ECTVs (i.e., not embedded within the other ECTV modalities)
Case-Based Discussion (CBD-ECTV)	Registrars prepare eight of their recent cases for an in-depth discussion with the ECT visitor. The visitor will choose at least four to discuss with the registrar via telephone or videoconference. Implemented as a standalone pandemic-mandated replacement for face-to-face or live video/telephone ECTVs (i.e., not embedded within the other ECTV modalities)

A copy of the questionnaire for registrars and ECT visitors is provided in Additional File 1. The questionnaire was developed by the research team, including individuals with subject matter expertise (senior medical educators with experience in the delivery and development of GP registrar education and formative assessment, including the design and delivery of CTVs (SG, RA, RB, LZ, PM) and individuals with expertise in quantitative questionnaire design and quantitative research methodology (AF, AT, MvD, LK, PM). The questionnaire and study methodology were piloted in a sub-sample of registrars and ECT visitors within one subregion of one RTO prior to the study taking place. The pilot evaluated feasibility, time to complete and overall acceptability of the questionnaire.

### Outcomes

The four primary outcomes were: 1) the perceived overall educational utility of the ECTV from the perspective of registrars; 2) the registrar’s self-reported likelihood to change their practice as a result of the feedback received from the ECT visitor; 3) registrar’s self-reported likelihood to change their training or learning as a result of the feedback from the ECT visitor; and 4) perceived overall educational utility of the ECTV from the perspective of the ECT visitor. These outcomes were elicited from the questionnaires using 5-point numeric rating scales with end points only labelled (e.g., 1 = Not at all useful/likely; 5 = Very useful/likely). Due to a strong positive-response ceiling effect, the outcome variables were dichotomised for multivariable analyses, combining ratings of 1 to 4 for comparison with ratings of 5, in other words, to explore the educational utility of ECTVs where the ratings were most useful/likely.

### Independent variables

A broad range of independent variables were included to maximise the exploratory potential of the study to identify characteristics of ECTVs associated with perceived educational utility, and to adjust for potential confounding in the relationship of ECTV modality and each of the outcome factors. Registrar and ECT visitor questionnaires elicited information about the registrar/ECT visitor, and details about the ECTV undertaken, including ECTV modality, number of patients seen/cases covered, content encountered during the visit, quality of feedback provided during the visit (with highest quality defined as being focused/specific, and easy to translate into action), frequency of feedback provided, consistency of feedback with that of their supervisor, and content discussed [21]. Of the 21 ‘content discussed’ items included in the questionnaire, 11 were a priori chosen for use as independent variables in multivariable analyses by two expert GPs with extensive clinical and medical education experience. The chosen topics/content items were consideration of the patient’s agenda; organisation and flow of the consultation; developing rapport; specific patient and/or contextual factors relevant to the consultation(s); time management; management planning; appropriate medications; appropriate investigations; patient follow-up; dealing with uncertainty; and physical examination.

To mitigate the potential impact of reduced ability to recall content details from the ECTV, any observations for which the time between the visit occurring and the questionnaire being completed exceeded 10 days were removed before analysis. Although the criterion cut-off was initially set in the protocol at more than two days after the visit [21], it was extended to 10 days to maximise availability of data for analysis, given challenges posed to data collection within the pandemic context.

### Statistical methods

Continuous variables were described using means and standard deviations for normally distributed data and median and interquartile range (IQR) for non-normally distributed data. Categorical variables were summarised as proportions.

Although some participants contributed data on multiple occasions, all analyses were cross-sectional. Univariable and multivariable associations of the four outcome factors were tested using logistic regression within the generalised estimating equations (GEE) framework to account for repeated measures within registrars and ECT visitors. An exchangeable working correlation structure was assumed. Covariates with a univariate p-value of < 0.20 were considered for inclusion in the multiple regression models.

Separate models were constructed for data elicited from ECT visitors and registrars. ECT visitor demographic variables were included in the registrar models, and registrar demographic variables were included in the ECT visitor models. Due to some missing demographic data (arising when ECT visitor/registrar demographics were not available for the corresponding ECTV occurrence), complete case (CC) and multiple imputation (MI) analyses were undertaken. CC was treated as the primary analysis and MI as the sensitivity analysis, to assess potential bias arising from CC analysis. Multiple imputation was performed for all missing independent variables, assuming data were missing at random. Imputation models included all independent variables and assumed an appropriate distribution for each variable. A total of 10 imputations were performed and estimates were combined using Rubin’s rules [23].

Significance was declared at the conventional 0.05 level, with the magnitude and precision of effect estimates also used to interpret results.

Analyses were programmed using STATA (versions 15.1 and 16.0) and SAS v9.84.

### Post-hoc analysis

Due to a strong effect of one registrar-specific variable (quality of feedback received during the visit), a post-hoc sensitivity analysis was run for each registrar model in which the quality of feedback variable was removed. These models were compared with complete case models to examine if the ‘quality of feedback’ effect was masking other less-salient effects in the models.

## Results

A total of 1811 invitations were sent to registrars (comprised of 818 individual registrars) and 1803 invitations were sent to ECT visitors (comprised of 248 individual ECT visitors). For registrars, 801 responses were received (response rate 41%; 453 individual registrars). For ECT visitors, 742 responses were received (response rate 39%; 189 individual ECT visitors).

The median time between the date of the visit and completion of the questionnaire was 3 days (IQR = 0–4) for registrars and 0 days (IQR = 0–3) for ECT visitors. After removing delayed questionnaire responses (registrars: n = 60; ECT visitors: n = 46), a total of 741 registrar observations and 696 ECT visitor observations were included in the analysis. Registrar and ECT visitor individual and practice characteristics are presented in Table 2.

**Table 2:** Characteristics of participating registrars and external clinical teaching (ECT) visitors

Registrar characteristics			ECT Visitor characteristics		
**Variable**	**Class**	**N (%)**	**Variable**	**Class**	**N (%)**
Gender	Female	433 (59.6)	Gender	Female	476 (72.2)
Age	Median (IQR)	32 (29–37)	Age	Median (IQR)	46 (36–55)
Regional Training Organisation	1	683 (92.2)	Regional Training Organisation	1	625 (89.6)
	2	27 (3.6)		2	43 (6.2)
	3	31 (4.2)		3	29 (4.2)
Country of primary medical qualification	Australia	528 (71.9)	Country of primary medical qualification	Australia	551 (82.7)
Fellowship (enrolled in)	RACGP	679 (91.6)	Fellowship (obtained)^a^	FRACGP	644 (92.5)
	FARGP	46 (6.2)		FARGP	44 (6.3)
	FACRRM	24 (3.2)		FACRRM	23 (3.3)
Training term	GPT1 GPT2 GPT3	277 (37.7) 324 (44.1) 133 (18.1)	Years since gaining fellowship	Median (IQR)	10 (4–22)
			Years of experience conducting ECTVs	Median (IQR)	4 (2–10)
			Experience as a medical educator	Currently	286 (42.9)
Training pathway	General	416 (56.6)		Previously	132 (19.8)
	Rural	319 (43.4)		N/A	248 (37.2)
			Experience as a GP supervisor	Currently	266 (39.9)
				Previously	126 (18.9)
Fulltime/part time training status	Full time	595 (81.1)		N/A	274 (41.1)
SEIFA-IRSD of current practice	Median (IQR)	6 (3–8)	Clinical practice hours per week	Mean (SD)	20.6 (10.7)
Rurality of current practice (MMM)	1	373 (50.3)	Rurality of current practice (ASGC-RA)	1	385 (57.7)
	2–3	190 (25.6)		2	133 (19.9)
	4–7	178 (24.0)		3–5	149 (22.3)

^a^percentages do not equal 100% as participants could select more than one option as applicable

FRACGP, Fellowship of the Royal Australian College of General Practitioners; FARGP, Fellowship in Advanced Rural General Practice (within RACGP); FACRRM, Fellowship of the Australian College of Rural and Remote Medicine; GPT, General practice training term; ECTVs, external clinical teaching visits; N/A, not applicable; MMM, Modified Monash Model classification of rurality (1 = major city to 7 = very remote) [41]; ASGC-RA, Australian Statistical Geographical Classification – Remoteness Area (1 = major city to 5-very remote) [42]; SEIFA-IRSD Socio Economic Index for Areas- Index of Relative Socioeconomic Disadvantage (1 = most disadvantage) [43]

Among registrar responses, 70.6% were for remotely conducted video/telephone ECTVs (*n* = 523) (95.8% of these were via video (*n* = 501) and 4.2% via telephone (*n* = 22)), 13.9% were for remote CNA-ECTVs (n = 103),1.7% were for CBD-ECTVs (*n* = 13) and 13.8% were for traditional face-to-face ECTVs (n = 102). For ECT visitor responses, 68.5% were for remote video/telephone ECTVs (n = 477) (94.8% of these were via video (*n* = 452) and 5.2% were via telephone (*n* = 25)), 15.7% were for CNA-ECTVs (n = 109), 2.4% were for CBD-ECTVs (n = 17) and 13.4% were for traditional face-to-face ECTVs (*n* = 93).

**Fig. 1:**
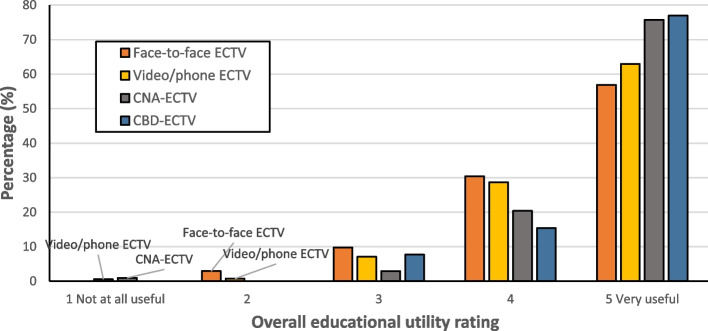
Registrar perceived educational utility ratings based on ECTV modality

**Fig. 2:**
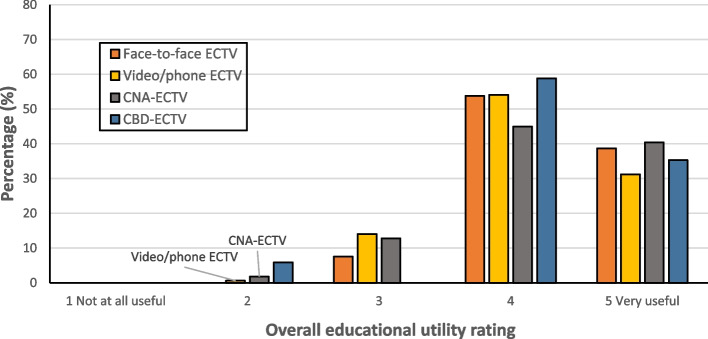
Clinical teaching visitor perceived educational utility ratings based on ECTV modality

Most registrars (64.1%, *n* = 475/741) rated the overall educational utility of ECTVs as ‘very useful’, and 58.5% (*n* = 429/734) and 47.9% (*n* = 352/735) of registrars rated their likelihood to change practice and their approach to learning/training as ‘very likely’, respectively. Fewer ECT visitors rated overall educational utility as ‘very useful’ (33.8%, *n* = 235/696). Detailed descriptive data for each outcome is presented in Table 3. Descriptive data for each outcome by ECTV modality is provided in Additional File 2, Supplementary Table 1. Descriptive comparisons of overall perceived educational utility by ECTV modality are presented in Fig. 1 (registrars) and Fig. 2 (ECT visitors)

**Table 3:** Perceived external clinical teaching visit utility ratings for each outcome

Outcome	Rating level	n	%
1. Registrar overall educational utility rating (*n* = 741) *‘Thinking about the ECTV overall, how educationally useful do you feel the ECTV was for you?’*	1 – Not at all useful	4	0.54
	2	7	0.94
	3	51	6.9
	4	204	27.5
	5 – Very useful	475	64.1
2. Registrar likelihood to change practice (*n* = 734) *‘How likely are you to change the way you practice as a result of the feedback you received from the CT visitor?’*	1 – Not at all likely	3	0.41
	2	17	2.32
	3	78	10.6
	4	207	28.2
	5 – Very likely	429	58.5
3. Registrar likelihood to change learning/training (*n* = 735) ‘*How likely are you to change your approach to learning or your training as a result of the feedback received from the CT visitor?’*	1 – Not at all likely	6	0.82
	2	28	3.81
	3	114	15.5
	4	235	32.0
	5 – Very likely	352	47.9
4. ECT Visitor overall educational utility rating (*n* = 696) *‘Thinking about the ECTV overall, how educationally useful do you feel the ECTV was for the registrar’*	1 – Not at all useful	-	-
	2	6	0.86
	3	88	12.6
	4	367	52.7
	5 – Very useful	235	33.8

### Associations of ECTV educational utility outcomes—registrars

Univariable comparisons of each independent variable against each outcome are presented in Additional File 2 (Supplementary Tables 2–5).

CNA-ECTVs were univariably associated with higher overall perceived educational utility ratings compared with face-to-face visits (odds ratio (OR) 1.93; 95% confidence interval (CI) 1.07 to 3.49). However, this did not remain significant in the multivariable model (Table 4). No significant differences in educational utility ratings were identified between face-to-face and remote video/phone ECTVs (Table 4).

**Table 4:** Factors associated with registrar ratings of overall educational utility of ECTVs (Outcome 1)^a^

		**Univariable**		**Multivariable**	
**Covariate**	**Class**	**OR [95% CI]**	**p**	**OR [95% CI]**	**p**
Type of Assessment	CBD ECTV	1.89 (0.64, 5.62)	.25	1.37 (0.26, 7.30)	.71
Referent: face-to-face ECTV	CNA ECTV	1.93 (1.07, 3.49)	.03	0.98 (0.41, 2.31)	.95
	Video/phone	1.21 (0.78, 1.86)	.39	0.98 (0.50, 1.93)	.95
Registrar AMG/IMG	IMG	1.39 (0.94, 2.06)	.10	1.32 (0.84, 2.09)	.23
RTO	RTO2	0.26 (0.10, 0.68)	.006	0.30 (0.11, 0.83)	.02
Referent: RTO1	RTO3	2.17 (0.87, 5.42)	.10	5.50 (1.54, 19.6)	.009
Overall quality of feedback	Rated 5	15.9 (10.5, 24.0)	<.001	12.8 (8.26, 19.9)	<.001
Consistency of feedback with supervisor	Rated 5	1.69 (1.26, 2.28)	<.001	1.04 (0.71, 1.53)	.84
Specific, meaningful feedback provided	3 or more cases	6.25 (3.26, 12.0)	<.001	3.13 (1.43, 6.87)	.004
Discussed consideration of patient’s agenda	Yes	1.43 (1.06, 1.92)	.02	1.00 (0.66, 1.52)	.99
Discussed organisation and flow	Yes	1.30 (0.96, 1.76)	.09	1.01 (0.67, 1.52)	.97
Discussed developing rapport	Yes	1.36 (1.04, 1.79)	.03	0.79 (0.52, 1.20)	.26
Discussed specific contextual factors	Yes	2.30 (1.63, 3.25)	<.001	1.48 (0.95, 2.32)	.09
Discussed management planning	Yes	1.94 (1.45, 2.58)	<.001	1.13 (0.77, 1.67)	.53
Discussed appropriate medications	Yes	1.69 (1.22, 2.34)	.002	1.05 (0.66, 1.66)	.84
Discussed appropriate investigations	Yes	1.49 (1.11, 2.00)	.007	1.15 (0.76, 1.73)	.51
Discussed patient follow-up	Yes	1.71 (1.26, 2.33)	<.001	1.10 (0.71, 1.70)	.68
Discussed dealing with uncertainty	Yes	1.90 (1.41, 2.56)	<.001	1.24 (0.84, 1.85)	.28
Discussed physical examination	Yes	1.52 (1.14, 2.02)	.004	1.05 (0.71, 1.56)	.81
Number of patients seen/cases discussed		0.92 (0.83, 1.02)	.10	0.91 (0.79, 1.05)	.22

^a^Outcome dichotomised as registrar overall utility ratings of 5 (n = 475/741, 64.1%) versus ratings of 1–4 (n = 266/741, 35.9%)

ECTV external clinical teaching visit; OR odds ratio; CI confidence interval; CBD case-based discussion; CNA case notes analysis; AMG Australian medical graduate; IMG international medical graduate; RTO regional training organisation

Other variables associated with overall educational utility ratings in multivariable analysis included: quality of feedback received (focused/specific, and easy to translate into action), OR: 12.8, 95 CI%: 8.26 to 19.9); and provision of specific/meaningful feedback for three or more consultations/cases (OR: 3.1, 95%CI: 1.43 to 6.87) (Table 4).

ECTV modality was univariably associated with the registrar’s rating of their likelihood to change practice (with CNA-ECTVs being rated as more likely to change when compared with face-to-face ECTVs). However, this was not reflected in the adjusted multivariable model (Table 5). Multivariable associations with registrar ratings of their likelihood to change their practice included: quality of feedback (OR: 2.5, 95%CI: 1.59 to 3.94); consistency of ECT visitor’s feedback with supervisor feedback (OR: 1.86, 95%CI: 1.25 to 2.77); provision of specific/meaningful feedback (OR: 2.7, 95%CI: 1.03 to 7.08); discussion of time management (OR: 1.7, 95%CI: 1.14 to 2.55); and discussion of appropriate investigations (OR: 1.57, 95%CI: 1.03 to 2.40) (Table 5).

**Table 5:** Factors associated with registrar ratings of likelihood to change practice based on feedback received during the ECTV (Outcome 2)^a^

		**Univariable**		**Multivariable**	
**Covariate**	**Class**	**OR [95% CI]**	**p**	**OR [95% CI]**	**p**
Type of Assessment	CBD ECTV	1.18 (0.39, 3.50)	.77	0.62 (0.08, 4.98)	.65
Referent: face-to-face	CNA ECTV	2.56 (1.44, 4.55)	.001	1.17 (0.42, 3.30)	.76
	Video/phone ECTV	1.30 (0.83, 2.05)	.26	0.98 (0.42, 2.30)	.96
Registrar AMG/IMG	IMG	1.85 (1.26, 2.71)	.002	1.81 (1.09, 3.01)	.02
RTO	RTO2	0.31 (0.12, 0.80)	.02	0.41 (0.15, 1.14)	.09
Referent: RTO1	RTO3	1.07 (0.49, 2.33)	.87	1.11 (0.21, 5.88)	.90
ECT Visitor gender	Female	1.33 (0.92, 1.92)	.13	1.30 (0.84, 2.01)	.23
ECT Visitor experience as ACRRM/RACGP examiner	Currently	1.34 (0.95, 1.89)	.09	1.28 (0.88, 1.87)	.19
Referent: N/A – no experience	Previously	1.43 (0.86, 2.36)	.16	1.80 (0.96, 3.37)	.07
Overall quality of feedback	Rated 5	3.88 (2.76, 5.45)	<.001	2.50 (1.59, 3.94)	<.001
Consistency of feedback with supervisor	Rated 5	2.09 (1.57, 2.79)	<.001	1.86 (1.25, 2.77)	.002
Registrar provided with opportunity to reflect on performance	3 or more cases	2.03 (1.20, 3.44)	.009	1.04 (0.45, 2.40)	.93
Specific, meaningful feedback provided	3 or more cases	3.42 (1.88, 6.24)	<.001	2.70 (1.03, 7.08)	.04
Discussed consideration of patient’s agenda	Yes	1.50 (1.11, 2.04)	.009	1.21 (0.76, 1.92)	.41
Discussed organisation and flow	Yes	1.33 (1.01, 1.75)	.04	0.98 (0.65, 1.49)	.94
Discussed developing rapport	Yes	1.22 (0.93, 1.61)	.15	0.85 (0.55, 1.30)	.44
Discussed specific contextual factors	Yes	1.42 (1.03, 1.97)	.03	1.00 (0.58, 1.73)	.99
Discussed time management	Yes	1.46 (1.12, 1.92)	.006	1.70 (1.14, 2.55)	.01
Discussed management planning	Yes	1.68 (1.27, 2.22)	<.001	1.12 (0.75, 1.67)	.59
Discussed appropriate medications	Yes	1.67 (1.25, 2.24)	<.001	1.14 (0.71, 1.82)	.59
Discussed appropriate investigations	Yes	1.62 (1.22, 2.17)	.001	1.57 (1.03, 2.40)	.04
Discussed patient follow-up	Yes	1.31 (0.98, 1.76)	.07	0.95 (0.62, 1.45)	.81
Discussed dealing with uncertainty	Yes	1.35 (1.01, 1.81)	.04	1.08 (0.71, 1.65)	.73
Discussed physical examination	Yes	1.33 (0.99, 1.78)	.06	1.07 (0.70, 1.65)	.76

^a^Outcome dichotomised as registrar perceived likelihood to change their practice ratings of 5 (n = 429/734, 58.5%) versus ratings of 1–4 (n = 305/734, 41.5%)

ECTV external clinical teaching visit; OR odds ratio; CI confidence interval; CBD case-based discussion; CNA case notes analysis; ECT external clinical teaching; ACRRM Australian College of Rural and Remote Medicine; RACGP Royal Australian College of General Practitioners; AMG Australian medical graduate; IMG international medical graduate; RTO regional training organisation

There was a significant univariable relationship between ECTV modality and the registrar’s rating of their likelihood to change their approach to training/learning (p = 0.04). However, this was not significant in multivariable analysis (all p > 0.27). Multivariable associations with registrar ratings of likelihood to change their approach to training/learning included: quality of feedback (OR: 3.19, 95%CI: 1.97 to 5.17); consistency of ECT visitor’s feedback with that of their supervisor (OR: 2.21, 95%CI: 1.46 to 3.34); and discussion of uncertainty (OR: 1.65, 95%CI: 1.08 to 2.51) (Table 6). Later training term was associated with lesser odds of changing approach to training/learning (registrar term GPT3, compared with GPT1, OR: 0.36, 95%CI: 0.19 to 0.70).

**Table 6:** Factors associated with registrar ratings of likelihood to change their approach to learning based on feedback received during the ECTV (Outcome 3)^a^

		**Univariable**		**Multivariable**	
**Covariate**	**Class**	**OR [95% CI]**	**p**	**OR [95% CI]**	**p**
Type of Assessment	CBD ECTV	0.89 (0.30, 2.60)	.83	0.24 (0.02, 2.98)	.27
Referent: face-to-face	CNA ECTV	2.09 (1.22, 3.56)	.007	1.52 (0.51, 4.51)	.45
	Video/phone ECTV	1.30 (0.85, 1.99)	.24	1.10 (0.46, 2.66)	.83
Registrar age		1.03 (1.00, 1.05)	.02	1.00 (0.96, 1.04)	.97
Registrar AMG/IMG	IMG	2.67 (1.83, 3.90)	<.001	3.12 (1.74, 5.59)	<.001
Registrar term	GPT 2	1.10 (0.83, 1.44)	.51	0.97 (0.66, 1.42)	.86
Referent GPT1	GPT 3	0.72 (0.47, 1.10)	.13	0.36 (0.19, 0.70)	.003
RTO	RTO2	0.27 (0.09, 0.83)	.02	0.37 (0.10, 1.36)	.14
Referent: RTO1	RTO3	1.41 (0.69, 2.89)	.35	2.24 (0.44, 11.3)	.33
SEIFA-IRSD of registrars’ practice		0.95 (0.90, 1.01)	.09	0.94 (0.87, 1.01)	.10
ECT Visitor AMG/IMG	IMG	1.39 (0.86, 2.26)	.18	1.36 (0.76, 2.41)	.30
ECT Visitor weekly clinical hours in general practice		1.01 (1.00, 1.03)	.16	1.02 (1.00, 1.04)	.12
Overall quality of feedback	Rated 5	4.42 (3.03, 6.44)	<.001	3.19 (1.97, 5.17)	<.001
Consistency of feedback with supervisor	Rated 5	2.32 (1.72, 3.12)	<.001	2.21 (1.46, 3.34)	<.001
Specific, meaningful feedback provided	3 or more cases	4.32 (2.13, 8.79)	<.001	1.62 (0.59, 4.43)	.35
Discussed consideration of patient’s agenda	Yes	1.32 (0.98, 1.79)	.07	0.95 (0.61, 1.48)	.81
Discussed developing rapport	Yes	1.25 (0.94, 1.65)	.12	0.89 (0.57, 1.40)	.62
Discussed specific contextual factors	Yes	1.79 (1.30, 2.46)	<.001	1.12 (0.68, 1.84)	.65
Discussed time management	Yes	1.38 (1.06, 1.79)	.02	1.35 (0.92, 1.96)	.13
Discussed management planning	Yes	1.81 (1.37, 2.40)	<.001	1.29 (0.84, 1.96)	.24
Discussed appropriate medications	Yes	1.84 (1.40, 2.43)	<.001	1.12 (0.71, 1.76)	.62
Discussed appropriate investigations	Yes	1.76 (1.35, 2.29)	<.001	1.48 (1.00, 2.20)	.05
Discussed dealing with uncertainty	Yes	1.62 (1.22, 2.15)	<.001	1.65 (1.08, 2.51)	.02
Discussed physical examination	Yes	1.48 (1.12, 1.96)	.005	1.00 (0.65, 1.53)	.99

^a^Outcome dichotomised as registrar perceived likelihood to change their learning ratings of 5 (*n* = 352/735, 47.9%) versus ratings of 1–4 (*n* = 383/735, 52.1%)

ECTV external clinical teaching visit; OR odds ratio, CI confidence interval; CBD case-based discussion; CNA case notes analysis; GPT general practice term; ECT external clinical teaching; AMG Australian medical graduate; IMG international medical graduate; RTO regional training organisation; SEIFA-IRSD Socio Economic Index for Areas- Index of Relative Socioeconomic Disadvantage (1 = most disadvantage) [43]

### Associations of ECTV educational utility outcomes – ECT visitors

Multivariable associations of ECT visitor educational utility ratings (“5-Very useful”) were the ECT visitor having previous experience as a GP supervisor (OR: 2.55, 95%CI: 1.27 to 5.13), ECT visitor gender (female, OR: 0.51, 95%CI: 0.29 to 0.91) and discussion of organisation and flow (OR: 1.53, 95%CI: 1.07 to 2.18) (Table 7). ECTV modality was not statistically significant at the *p* < 0.05 level in the univariable or multivariable ECT visitor perceived educational utility analyses, though there was some evidence (OR 0.55, 95%CI 0.29 to 1.04; *p* = 0.07) for visitors rating video/phone CTVs as of less utility than face-to-face CTVs).

**Table 7:** Factors associated with ECT visitor ratings of overall educational utility of ECTVs (Outcome 4)^a^

		**Univariable**		**Multivariable**	
**Covariate**	**Class**	**OR [95% CI]**	**p**	**OR [95% CI]**	**p**
Type of Assessment	CBD ECTV	0.66 (0.19, 2.32)	.52	0.61 (0.17, 2.22)	.46
Referent: face-to-face	CNA ECTV	1.02 (0.51, 2.03)	.95	0.97 (0.42, 2.22)	.94
	Video/phone ECTV	0.63 (0.39, 1.03)	.07	0.55 (0.29, 1.04)	.07
Rurality of ECT Visitor practice	Inner regional	0.57 (0.32, 1.00)	.05	0.68 (0.36, 1.28)	.23
Referent: Major City	Outer regional	0.77 (0.45, 1.32)	.34	0.64 (0.33, 1.22)	.17
ECT Visitor experience as medical educator	Currently	0.78 (0.49, 1.24)	.29	0.89 (0.52, 1.53)	.68
Referent: N/A – no experience	Previously	0.50 (0.23, 1.08)	.08	0.62 (0.28, 1.35)	.23
ECT Visitor experience as GP Supervisor	Currently	1.34 (0.83, 2.17)	.24	1.22 (0.72, 2.07)	.47
Referent: N/A – no experience	Previously	2.12 (1.12, 4.00)	.02	2.55 (1.27, 5.13)	.009
ECT Visitor experience as ACRRM/RACGP examiner	Currently	1.43 (0.90, 2.28)	.13	1.49 (0.89, 2.48)	.13
Referent: N/A – no experience	Previously	0.78 (0.35, 1.75)	.55	0.59 (0.28, 1.27)	.18
ECT Visitor gender	Female	0.60 (0.37, 0.98)	.04	0.51 (0.29, 0.91)	.02
RTO	RTO2	0.53 (0.24, 1.20)	.13	0.70 (0.27, 1.79)	.46
Referent: RTO1	RTO3	1.83 (0.78, 4.26)	.16	1.07 (0.31, 3.69)	.91
SEIFA-IRSD of registrars’ practice		1.04 (0.99, 1.10)	.11	1.06 (1.00, 1.12)	.06
Registrar able to reflect on performance	3 or more cases	2.07 (1.32, 3.25)	.002	1.60 (0.97, 2.65)	.07
Specific, meaningful feedback provided	3 or more cases	1.92 (1.08, 3.41)	.03	1.16 (0.57, 2.37)	.68
Discussed consideration of patient’s agenda	Yes	1.40 (1.01, 1.93)	.04	1.31 (0.90, 1.92)	.16
Discussed organisation and flow	Yes	1.57 (1.15, 2.15)	.004	1.53 (1.07, 2.18)	.02
Discussed management planning	Yes	1.52 (1.15, 2.02)	.003	1.39 (0.97, 1.99)	.07
Discussed appropriate medications	Yes	1.36 (0.97, 1.93)	.08	1.12 (0.75, 1.67)	.58
Discussed appropriate investigations	Yes	1.33 (0.99, 1.77)	.06	1.22 (0.86, 1.72)	.26
Discussed patient follow-up	Yes	1.37 (0.98, 1.93)	.07	0.94 (0.64, 1.40)	.78
Discussed physical examination	Yes	1.41 (1.07, 1.86)	.02	1.30 (0.95, 1.78)	.10

^a^Outcome dichotomised as ECT visitor overall utility ratings of 5 (n = 235/696, 33.8%) versus ratings of 1–4 (n = 461/696, 66.2%)

ECTV external clinical teaching visit; OR odds ratio; CI confidence interval; CBD case-based discussion; CNA case notes analysis; ECT clinical teaching; ACRRM Australian College of Rural and Remote Medicine; RACGP Royal Australian College of General Practitioners; RTO regional training organisation; SEIFA-IRSD Socio Economic Index for Areas- Index of Relative Socioeconomic Disadvantage (1 = most disadvantage) [43]

Findings from multiple imputation (Additional File 2, Supplementary Tables 6–9) and post-hoc sensitivity analysis models (omitting quality of feedback provided, Supplementary Tables 10–13) demonstrated minor variations in associations between independent variables and outcomes. However, the overall findings were not substantially different from those seen in the complete case analysis models.

## Discussion

### Summary of main findings

This study found that ECTVs were widely perceived by registrars and ECT visitors to be of high educational utility. Registrars also generally perceived ECTVs as having a high likelihood of influencing changes in their practice and in their approach to learning. Collectively this supports the overall educational utility of ECTVs as a WBA in vocational general practice training.

In multivariable analysis, we found no statistically significant differences in perceived educational utility of remote ECTV modalities when compared with face-to-face visits. This observation was consistent for both registrar- and ECT visitor-reported outcomes. There was some evidence (p = 0.07 on multivariable analysis), however, for ECT visitors rating video/phone ECTVs less favourably than face-to-face ECTVs.

Several features of feedback provided during ECTVs were significantly associated with registrar ratings of educational utility. The most striking finding was for the effect size regarding quality of feedback provided, where the odds of registrars rating the educational utility of the visits as ‘very useful’ were 12.8 times higher if the quality of the feedback provided was also rated as’very useful’. This was similarly reflected in likelihood of changing practice and approach to training/learning outcomes, albeit with more modest effect sizes (multivariable ORs of 2.5 and 3.2 respectively). More frequent provision of feedback during the visit (i.e., for three or more consultations/cases), and the consistency of the ECT visitor’s feedback with that previously given by the registrar’s supervisor, were also significantly associated with registrar-rated educational utility outcomes.

ECT visitor perceptions of the educational utility of ECTVs were generally consistent with registrar perceptions. However, there were some differences in ECTV content-related independent variables associated with utility ratings when compared with registrars (for example, the specific topics discussed).

### Comparison with previous literature

To our knowledge, this is the first quantitative study to examine the perceived educational utility and associations of utility ratings for ECTVs, inclusive of both traditional face-to-face and remote ECTV modalities. A recent Australian qualitative study found that general practice trainees and assessors value the educational opportunities offered by ECTV direct observations and highlighted the importance of feedback conversations between consultations during the visit [17]. Our findings, about educational utility and associations of feedback provided during ECTVs, triangulate well with the observations of Sturman et al. [17] Our results about frequency of feedback also align with the findings of an earlier qualitative study investigating the value of adding RCA to direct observation assessments, which suggested that observation and feedback provision for three or more consultations is required for generating learning [16]. Our results regarding the educational utility of CNA-ECTVs, which share many similarities to RCAs conducted with direct observation, also support the conclusions of Ingham et al., where RCAs were viewed as having utility for learning within the direct observation session [16].

While we are not aware of any previous studies that have similarly assessed educational utility of face-to-face and remote formative assessment modalities within the GP training setting, there is some evidence regarding formative assessment conducted using videotaped sessions of registrar consultations [24, 25]. Although our findings are not directly comparable with these earlier studies, which did not involve real-time videoconferencing or teleconferencing, collectively the findings of these studies support a role for remote observation of practice for formative purposes. A recent systematic review of the feasibility and acceptability of remote clinical assessments in a variety of clinical settings, including general practice, similarly indicated overall in-principle support for the potential for remote assessments for summative and formative purposes [26].

Within existing medical education formative feedback theory literature, face-to-face discussion of feedback is regarded as one of the fundamental principles of effective feedback [27]. The apparent lack of difference in perceived educational utility outcomes between face-to-face and remote modalities in our study fails to support this.

Our findings about features of the feedback received (including receiving feedback for three or more consultations/cases, receiving feedback that is specific and actionable, and consistency of the feedback received with that of the supervisor) being positively associated with all three registrar educational utility outcomes, is consistent with the inherent link between feedback and achieving the formative aims of WBA [28, 29]. Effective feedback has been widely considered in medical education WBA contexts, and while there are many acknowledged complexities in what constitutes effective feedback [30], our findings empirically support the value of feedback that is interactive, timely and specific [31, 32].

Our multivariable findings are also consistent with education feedback theory, which asserts that it is the nature, content and collaborative discussion of feedback that drives its formative utility [27, 33]. Van de Vleuten et al. emphasise, in their theory-based framework for programmatic assessment, that feedback effectiveness is a function of the user (i.e., assessor and learner), rather than the instrument/s utilised. [11]. Our study provides empirical evidence to support the applicability of this theory within the direct observation WBA context in general practice training, where quality feedback was perceived as useful regardless of the ECTV modality by which the feedback was delivered.

Our findings also support the robustness of Pelgrim’s assessment feedback process schema, which identifies feedback content and ‘delivery’ as the central element of useful feedback obtainment, while noting a need for more exploration of further external/contextual variables [20]. Our observation that the features of the feedback received were most strongly related to perceived educational utility outcomes than a diversity of other situational/contextual factors, supports that contextual variables are a less prominent feature than the feedback itself within an effective feedback process model.

A specific novel aspect of our findings, not well-explored in existing literature, is that the non-direct observation formats (CBD-ECTVs and CNA-ECTVs) appear to provide a similarly useful substrate for educationally useful feedback when compared with direct observation ECTV formats.

### Implications for practice, policy, and future research

Our findings support the assertion that ECTVs have a high level of perceived educational utility, affirming their ongoing inclusion as a fundamental element of formative assessment frameworks within Australian vocational general practice training. Our findings on ECTVs are likely transferable to other medical training programs internationally that use similar formative direct observation within their assessment frameworks. Implementation of ECTV-style assessments may be of interest for other medical education programs as a means of increasing the frequency of direct observation, which is emphasised as critical for optimising competency-based medical education [34]. We also suggest that in the context of formative WBA, non-direct observation modalities may offer a similarly viable substrate for useful, actionable feedback for learning, which is also likely to be generalisable across different medical education WBA training contexts.

The similarities of perceived educational utility across different ECTV modalities provides initial support for inclusion of remote ECTV modalities within post-pandemic WBA frameworks. Potentially, the number of traditional face-to-face ECTVs could be reduced by substituting in some remote ECTV modalities, without detriment to the overall educational value for the registrar. A ‘hybrid’ ECTV delivery model, combining a mixture of face-to-face and remote ECTVs across the course of training, may capitalise on the benefits of both assessment modalities, including resource optimisation, convenience, and varied learning opportunities. Furthermore, through inclusion of remote ECTV modalities, it may be possible to offer additional ECTVs without imposing substantive additional resourcing burden across a registrar’s course of training.

It must be acknowledged, however, that the different types of ‘remote ECTV’ that were included in the study ranged from real-time, videoconference, ECT visitor direct observation of patient consultations, through to telephone-based discussion of previous cases selected by the registrar. Although we did not find a difference in overall perceived educational utility of different assessment modalities, the inherent differences between these remote formats and that of traditional face-to-face direct observation ECTVs, suggest potential for differences in ‘educational richness’ that were not captured in the present study. Further research is required to explore how different context-specific learning experiences may vary by modality. For example, opportunities for observation of performing physical examination has been previously identified as a limitation of remote assessment modalities [2, 26]. Further investigation of the benefits and limitations of different modalities is required to gain a better understanding the relative merits and limitations of different ECTV modalities.

Given the salient relationship between feedback quality and educational utility of ECTVs, it is essential that ECTV training and implementation (for both registrars and ECT visitors) incorporates evidence-based principles of feedback, including specificity, actionability, timeliness, two-way/collaborative, and underpinned by mutual trust and credibility [27]. This is particularly important for remote modalities, where video- or telephone-based observations may present additional challenges in fostering the personal connection necessary for effective feedback [35, 36].

The association we identified for consistency of feedback from the ECT visitor with that of the supervisor (where registrars perceived this feedback to be of more utility) is an interesting consideration for ECTVs, where the implicit intent of the visit is to obtain feedback from an independent, experienced, and objective GP assessor. While consistency of feedback across different assessors may enhance its perceived trustworthiness, our findings raise an interesting question about registrars’ potential uptake of feedback from the ECTV in cases where it is inconsistent with that of their supervisor. This suggests a need for registrars to be supported in developing ‘feedback literacy’, defined as ‘appreciating feedback, making judgments, managing affect, and taking action’ [37, 38], to help maximise learning potential of the ECTV.

Differences in associations of educational utility ratings between our registrar and ECT visitor analyses may reflect ECT visitors and registrars having different priorities for learning within the ECTV, with ECT visitors placing greater emphasis on more holistic aspects of professional practice. These different priorities and perspectives should be considered in the feedback conversations that occur during ECTVs.

Our quantitative study provides foundational insight into the educational utility of different ECTV modalities, emphasising the key role of feedback within these WBAs. Further qualitative research is required to explore the barriers and enablers of effective feedback within ECTVs, and to gain a deeper understanding of the interplay between substrates of educational richness, feedback, and practice change.

### Strengths and limitations

The strengths of this study include a large sample size, and a reasonable response rate for studies of GP clinicians [39], particularly notable given pandemic conditions. The study included participants from a variety of practice settings across multiple RTOs (encompassing urban, rural, and remote training settings, and diverse socio-economic profiles) and included GP registrars across different stages of training. Perceptions of both registrars and ECT visitors provided multiple perspectives. Many clinically and educationally relevant independent variables were included as covariates in analyses, enabling comprehensive consideration of factors that may relate to perceived educational utility of ECTVs.

The pandemic-driven shift to remote ECTV delivery concurrent with the study provided the opportunity to assess perceived educational utility of both face-to-face and remote ECTV modalities. However, the potential effects of the pandemic context of data collection on the generalisability of the findings should be acknowledged. It is possible that the pandemic setting may have impacted upon registrars’ and ECT visitors’ judgements about how educationally useful the ECTV was, although the potential direction of this bias is unknown.

Other limitations are noteworthy. First, the cross-sectional nature of the analyses precludes inference of causality despite several strong relationships identified. Second, we used registrars’ self-report for the likelihood of changing their practice and training/learning behaviours. Practical and resourcing constraints precluded objective measurement of actual behaviour change in this study and we were unable to explore the intention-behaviour gap. Despite inherent limitations of using self-reported intention, there is evidence to support intention as a valid proxy measure for behavioural change [40]. Third, there was a large ceiling effect for the utility and likelihood to change outcomes, necessitating grouping ratings of one to four, for comparison with the highest rating of five. This could have impacted our ability to detect variability between the different ECTV modalities.

Fourth, a registrar’s previous exposure to a face-to-face ECTV could have had an impact on their ratings of subsequent remote ECTVs. It is possible that some registrars participating in the study had not previously experienced a face-to-face ECTV and therefore had no criteria to contrast remote ECTVs against. Finally, information technology (IT) issues were not specifically addressed in the questionnaire. Such logistical/practical issues have been acknowledged as problematic for remote assessment [26], and must be considered and addressed in the future rollout of remote ECTV modalities.

## Conclusion

Our findings indicate that ECTVs are perceived as highly educationally useful as a direct observation-based formative WBA. Registrars and ECT visitors rated highly face-to-face and remote ECTV modalities for perceived educational utility. The quality and features of feedback appear to be the most important factors in ECTVs as an assessment for learning. The perceived educational utility of remote ECTVs provides support for inclusion of remote ECTVs within WBA frameworks in vocational general practice training.

## Supplementary Information

Supplementary Material 1.

Supplementary Material 2.

## Data Availability

The datasets used for this study are available from the corresponding author upon reasonable request.

## Abbreviations

ACRRM: Australian College of Rural and Remote Medicine
AMG: Australian medical graduate
ASGC-RA: Australian Statistical Geographical Classification – Remoteness Area
CBD: Case-based discussion (ECTV)
CC: Complete case
CI: Confidence interval
CNA: Case notes analysis (ECTV)
ECT visitor: External clinical teaching visitor
ECTV: External clinical teaching visit
FACRRM: Fellowship of the Australian College of Rural and Remote Medicine
FARGP: Fellowship in Advanced Rural General Practice (within RACGP)
FRACGP: Fellowship of the Royal Australian College of General Practitioners
GEE: Generalised estimating equations
GP: General practitioner
GPT: General practice term
GPTT: General Practice Training Tasmania (RTO)
IMG: International medical graduate
IQR: Interquartile range
IT: Information technology
MI: Multiple imputation
MMM: Modified Monash Model
N/A: Not applicable
NTGPE: Northern Territory General Practice Education (RTO)
OR: Odds ratio
RACGP: Royal Australian College of General Practitioners
RCA: Random case analysis
RTO: Regional training organisation
SEIFA-IRSD: Socio Economic Index for Areas- Index of Relative Socioeconomic Disadvantage
WBA: Workplace-based assessment
