# Supplementary material for: Educational utility of observational workplace-based assessment modalities in Australian vocational general practice training: a cross-sectional study
Source: BMC Med Educ. 2025 May 23;25:762. doi: 10.1186/s12909-025-07328-y (PMC12102905; doi:10.1186/s12909-025-07328-y)
Supplement: Supplementary file 1 — Supplementary Material 1. [file 12909_2025_7328_MOESM1_ESM.pdf]

Additional File – Questionnaires

Registrar Questionnaire

Questionnaire notes:

- For questionnaires administered within RTO2 and RTO3, the term 'Clinical Teaching Visit' and abbreviation 'CTV' is replaced with 'external Clinical Teaching Visit' / 'ECTV' in line with terminology used within these organisations.
- Variations in questions for different RTOs (arising from variations in CTV formats and terminologies used between training organisations) are indicated using the 'questionnaire allocation' and 'RTO-questionnaire allocations' columns
- As face-to-face CTVs were suspended due to COVID-19, RTO1 implemented three remote alternatives: a remote CTV session via video conferencing or telephone, a remote random case analysis session (RCA-CTV), and a remote case-based discussion session (CBD-CTV) – these are denoted and colour-coded in the master questionnaire
- Skip logic in the online questionnaire was used to deliver the correct set of questions for different modalities

Question item	Questionnaire allocation	RTO-questionnaire allocation	Question	Response options			
1	Questions allocated under the following categories:<br>"Face-to-face CTV"<br>"Video/phone CTV"<br>'RCA-CTV'<br>'CBD-CTV'	RTO1 only	Please indicate which CTV assessment you completed<br><br>Please note:<br>*Face-to-face CTV:*<br>CT visitor is present while you see patients at your practice. May include telehealth consultations, random case analyses or simulated cases (role plays).<br>*Video/phone CTV:*<br>CT visitor is present via Zoom/phone while you see patients either face-to-face and/or via telehealth. May include simulated cases (role plays).<br>*RCA-CTV:*<br>CT visitor conducts a series of Random Case Analyses only with you via Zoom/phone.<br>No patient consults are observed.	◯ Face-to-face CTV	◯ Video/phone CTV	◯ Random Case Analysis (RCA)-CTV	◯ Case Based Discussion (CBD)-CTV

			CBD-CTV:<br>CT visitor conducts a series of Case-Based Discussions only with you via Zoom/phone. No patient consults are observed.	
2	Video/phone CTV **only**	RTO1 only	How was the CTV conducted?	◯ By video ◯ By telephone
	RCA-CTV		How was the RCA-CTV conducted?	◯ By video ◯ By telephone
	CBD-CTV		How was the CBD-CTV conducted?	◯ By video ◯ By telephone
	Face-to-face CTV **and** Video/phone	RTO2 and RTO3	How was the CTV conducted?	◯ In person (face-to-face) ◯ By video ◯ By telephone
3	Face-to-face CTV **and** Video/phone	All RTOs	In what session was the CTV held?	◯ Morning ◯ Afternoon
4	Face-to-face CTV **and** Video/phone	ALL RTOs	How many patients did you see during the CTV?	___________________________________
	RCA-CTV	RTO1 only	How many cases did you discuss during the RCA-CTV	___________________________________
	CBD-CTV		How many cases did you discuss during the CBD-CTV	___________________________________
5	Face-to-face CTV **and** Video/phone	All RTOs	How many of these were telehealth consultations?	___________________________________
6	Face-to-face **and** video/phone	RTO1 only	Did the visit include any simulated cases?	◯ No ◯Yes (how many?) _____
7	Face-to-face CTV **and** Video/phone	All RTOs	Thinking about the CTV overall, how educationally useful do you feel the CTV was for you?	◯ 1 - Not at all useful ◯ 2 ◯ 3 ◯ 4 ◯ 5 - Very useful

	RCA-CTV	RTO1 only	Thinking about the RCA-CTV overall, how educationally useful do you feel the RCA-CTV was for you?	◯ 1 - Not at all useful ◯ 2 ◯ 3 ◯ 4 ◯ 5 - Very useful
	CBD-CTV		Thinking about the CBD-CTV overall, how educationally useful do you feel the CBD-CTV was for you?	◯ 1 - Not at all useful ◯ 2 ◯ 3 ◯ 4 ◯ 5 - Very useful
8	Face-to-face CTV **and** Video/phone	RTO1 and RTO 2	Do you feel the presence of the CT visitor affected how you performed any clinical tasks compared to your usual practice?	◯ Not at all ◯ Yes, a little ◯ Yes, a lot If yes, how (optional): ___
		RTO3	Do you feel the presence of the ECT visitor/ECTV recording affected how you performed any clinical tasks compared to your usual practice?	
9	Face-to-face CTV **and** Video/phone	ALL RTOs	Did you consult your supervisor in any way <u>(including: face-to-face, phone, electronic messaging)</u> during the CTV?	◯ Yes ◯ No
10	Face-to-face CTV **and** Video/phone	ALL RTOs	During this CTV did the CT visitor conduct a Random Case Analysis (RCA) with you? *An RCA is a specific method of case note review where past records are selected at <u>random</u> by the CT visitor, not by you.*	◯ No ◯Yes (how many?) ________
11	Face-to-face CTV **and** Video/phone	ALL RTOs	Of the following, which activities occurred during this CTV?	
	RCA-CTV	RTO1 only	Of the following, which activities occurred during this RCA-CTV?	
	CBD-CTV		Of the following, which activities occurred during this CBD-CTV?	
	Face-to-face CTV **and** Video/phone	ALL RTOs	a. The CT visitor gave me the opportunity to reflect on my performance.	◯ Not for any consultation ◯ Yes, for 1 consultation ◯ Yes, for 2 consultations ◯ Yes, for 3 or more consultations
	RCA-CTV	RTO1 only	a. The CT visitor gave me the opportunity to reflect on my performance.	◯ Not for any case ◯ Yes, for 1 case ◯ Yes, for 2 cases ◯ Yes, for 3 cases

	CBD-CTV		a. The CT visitor gave me the opportunity to reflect on my performance.	◯ Not for any case	◯ Yes, for 1 case	◯ Yes, for 2 cases	◯ Yes, for 3 or more cases
	Face-to-face CTV **and** Video/phone	ALL RTOs	b. The CT visitor provided me with meaningful feedback on a specific aspect of my performance.	◯ Not for any consultation	◯ Yes, for 1 consultation	◯ Yes, for 2 consultations	◯ Yes, for 3 or more consultations
	RCA-CTV	RTO1 only	b. The CT visitor provided me with meaningful feedback on a specific aspect of my performance.	◯ Not for any case	◯ Yes, for 1 case	◯ Yes, for 2 cases	◯ Yes, for 3 or more cases
	CBD-CTV		b. The CT visitor provided me with meaningful feedback on a specific aspect of my performance.	◯ Not for any case	◯ Yes, for 1 case	◯ Yes, for 2 cases	◯ Yes, for 3 or more cases
	Face-to-face CTV **and** Video/phone	ALL RTOs	c. The CT visitor <u>observed</u> me performing a physical examination (other than BP and pulse measurement).	◯ Not for any consultation	◯ Yes, for 1 consultation	◯ Yes, for 2 consultations	◯ Yes, for 3 or more consultations
12	Face-to-face CTV **and** Video/phone	ALL RTOs	Regarding the consultations observed, which of the following areas (if any) did the CT visitor <u>discuss</u> with you? Please select all that apply:	□ Identifying the reason for the consultation<br>□ Exploration of patient's problems<br>□ Consideration of the patient's agenda<br>□ Organisation and flow<br>□ Non-verbal behaviour<br>□ Developing rapport<br>□ Appropriateness of physical examination components performed/proposed<br>□Physical examination technique<br>□ Diagnosis<br>□ Explanation of diagnosis to patient<br>□ Specific patient and/or location contextual factors relevant to the consultation(s)<br>(*e.g. rural/remote, socioeconomic, Aboriginal and/or Torres Strait*		□ Time management<br>□ Management planning<br>□ Appropriate medications<br>□ Appropriate investigations<br>□ Medication prescribing processes<br>□ Documentation in patients' medical records<br>□ Referrals<br>□ Patient follow-up<br>□ Dealing with uncertainty<br>□ Safety netting<br>*(Contingency planning with the patient to provide a management strategy for a change in symptoms, including explicit instruction for action(s) given specific circumstances)*	
Note for item 12, response options are the same across all formats. Variation in question terminology only.	RCA-CTV	RTO1 only	Regarding the cases reviewed, which of the following areas (if any) did the CT visitor <u>discuss</u> with you? Please select all that apply:				
	CBD-CTV		Regarding the cases reviewed, which of the following areas (if any) did the CT visitor <u>discuss</u> with you? Please select all that apply:				

				Islander, non-English speaking background factors)
13	Face-to-face CTV **and** Video/phone	ALL RTOs	Thinking about the feedback you received in the CTV session, how would you classify the overall quality of the feedback?	○ 1 - Broad, non-specific; difficult for me to translate into action ○ 2 ○ 3 ○ 4 ○ 5 - focused/specific; easy for me to translate into action
	RCA-CTV	RTO1 only	Thinking about the feedback you received in the RCA-CTV session, how would classify the overall quality of the feedback?	○ 1 - Broad, non-specific; difficult for me to translate into action ○ 2 ○ 3 ○ 4 ○ 5 - focused/specific; easy for me to translate into action
	CBD-CTV		Thinking about the feedback you received in the CBD-CTV session, how would classify the overall quality of the feedback?	○ 1 - Broad, non-specific; difficult for me to translate into action ○ 2 ○ 3 ○ 4 ○ 5 - focused/specific; easy for me to translate into action
14	All	ALL RTOs	How consistent did you find CT visitor feedback with the feedback you have previously received from your supervisor?	○ 1 – not at all consistent ○ 2 ○ 3 ○ 4 ○ 5 – very consistent ○ I didn't receive any feedback
15	All	ALL RTOs	How likely are you to change the way you practice as a result of the feedback you received from the CT visitor?	○ 1 – not at all likely ○ 2 ○ 3 ○ 4 ○ 5 – very likely ○ I didn't receive any feedback
16	All	ALL RTOs	How likely are you to change your approach to learning or your training as a result of the feedback you received from the CT visitor?	○ 1 – not at all likely ○ 2 ○ 3 ○ 4 ○ 5 – very likely ○ I didn't receive any feedback
17	Both Face-to-face CTV **and** Video/phone	ALL RTOs	Is there anything else you would like to comment on regarding the content, educational usefulness, or any other aspect of the CTV?	________________________________
	RCA-CTV	RTO1 only	Is there anything else you would like to comment on regarding the content, educational usefulness, or any other aspect of the RCA-CTV?	________________________________

	CBD-CTV		Is there anything else you would like to comment on regarding the content, educational usefulness, or any other aspect of the CBD-CTV?	________________________________
18	All	ALL RTOs	What best describes your gender?	◯ Male ◯ Female ◯ Prefer to self-describe as:___________ (optional) ◯ Prefer not to say
19	All	ALL RTOs	Your age	________________________________
20	All	ALL RTOs	Where did you qualify as a doctor (primary medical degree)?	◯ Australia ◯ Other, please specify _____________________
21	All	ALL RTOs	Which specialist GP Fellowship are you working towards? (tick all that are applicable)	☐ FRACGP ☐ FARGP ☐ ACRRM
22	All	ALL RTOs	Regarding your current training, are you enrolled	◯ Full-time ◯ Part-time
23	All	ALL RTOs	Which training term are you doing now?	◯ GPT1 and/or PRR1 ◯ GPT2 and/or PRR2 ◯ GPT3 and/or PRR3 ◯ Other, please specify ____________
24	All	RTO1 only	Which pathway are you currently enrolled in?	◯ General ◯ Rural
25	All	ALL RTOs	How many GPs (full time equivalents) work with you at the practice where your CTV was conducted?	◯ <2 ◯ 2-4 ◯ 5-9 ◯ >10
26	All	ALL RTOs	How many hours do you work each week as a GP?<br>*Include only face-to-face, rostered, patient consultation time*	______________________________________

CT Visitor Questionnaire

Questionnaire notes:

- For questionnaires administered within RTO2 and RTO3, the term 'Clinical Teaching Visit' and abbreviation 'CTV' is replaced with 'external Clinical Teaching Visit' / 'ECTV' in line with terminology used within these organisations.
- Variations in questions for different RTOs (arising from variations in CTV formats and terminologies used between training organisations) are indicated using the 'questionnaire allocation' and 'RTO-questionnaire allocations' columns
- As face-to-face CTVs were suspended due to COVID-19, RTO1 implemented three remote alternatives: a remote CTV session via video conferencing or telephone, a remote random case analysis session (RCA-CTV), and a remote case-based discussion session (CBD-CTV) – these are denoted and colour-coded in the master questionnaire
- Skip logic in the online questionnaire was used to deliver the correct set of questions for different modalities

Question item	Questionnaire allocation	RTO-questionnaire allocation	Question	Response options			
1	Denoted as belonging to the following questionnaires 'Live CTV' 'RCA-CTV' 'CBD-CTV'	RTO1 only	Please indicate which CTV assessment you conducted with the registrar.<br><br>Please note:<br>*Face-to-face CTV:*<br>CT visitor is present while the registrar sees patients at their practice. May include telehealth consultations, random case analyses or simulated cases (role plays).<br>*Video/phone CTV:*<br>CT visitor is present via Zoom/phone while the registrar sees patients either face-to-face and/or via telehealth. May include simulated cases (role plays).<br>*RCA-CTV:*<br>CT visitor conducts a series of Random Case Analyses only with the registrar via Zoom/phone. No patient consults are observed.<br>*CBD-CTV:*	◯ Face-to-face CTV	◯Video/phone CTV	◯ Random Case Analysis (RCA)-CTV	◯ Case Based Discussion (CBD)-CTV

			CT visitor conducts a series of Case-Based Discussions only with the registrar via Zoom/phone. No patient consults are observed.	
2	Video/phone **only**	RTO1 only	How was the CTV conducted?	◯ By video ◯ By telephone
	RCA-CTV		How was the RCA-CTV conducted?	◯ By video ◯ By telephone
	CBD-CTV		How was the CBD-CTV conducted?	◯ By video ◯ By telephone
	Face-to-face CTV **and** Video/phone	RTO2 and RTO3	How was the CTV conducted?	◯ In person (Face-to-Face) ◯ By video ◯ By telephone
3	Face-to-face CTV **and** Video/phone	All RTOs	In what session was the CTV held?	◯ Morning ◯ Afternoon
4	Face-to-face CTV **and** Video/phone	ALL RTOs	How many patients did the registrar see during the CTV?	________________________________
	RCA-CTV	RTO1 only	How many cases did you discuss with the registrar during the RCA-CTV	________________________________
	CBD-CTV		How many cases did you discuss with the registrar during the CBD-CTV	________________________________
5	Face to face CTV **and** Video/phone	ALL RTOs	How many of these were telehealth consultations?	________________________________
6	Face to face CTV **and** Video/phone	RTO1	Did the visit include any simulated cases?	◯ No ◯ Yes (how many?) _____
7	Face-to-face CTV **and** Video/phone	All RTOs	Thinking about the CTV overall, how educationally useful do you feel the CTV was for the registrar?	◯ 1 - Not at all useful ◯ 2 ◯ 3 ◯ 4 ◯ 5 - Very useful

	RCA-CTV	RTO1 only	Thinking about the RCA-CTV overall, how educationally useful do you feel the RCA -CTV was for the registrar?	◯ 1 - Not at all useful	◯ 2	◯ 3	◯ 4	◯ 5 - Very useful
	CBD-CTV		Thinking about the CBD-CTV overall, how educationally useful do you feel the CBD-CTV was for the registrar?	◯ 1 - Not at all useful	◯ 2	◯ 3	◯ 4	◯ 5 - Very useful
8	Face-to-face CTV **and** Video/phone	ALL RTOs	Did the registrar consult with their supervisor in any way <u>(including: face-to-face, phone, electronic messaging)</u> during the CTV?	◯ Yes		◯ No		
9	Face-to-face CTV **and** Video/phone	ALL RTOs	During the CTV did you conduct a Random Case Analysis (RCA) with the registrar? *An RCA is a specific method of case note review where past records are selected at <u>random</u> by you, not the registrar.*	◯ No		Yes (how many?) ________		
10	Face-to-face CTV **and** Video/phone	ALL RTOs	Of the following, which activities occurred during this CTV?					
	RCA-CTV	RTO1 only	Of the following, which activities occurred during this RCA-CTV?					
	CBD-CTV		Of the following, which activities occurred during this CBD-CTV?					
	Face-to-face CTV **and** Video/phone	ALL RTOs	a. I gave the registrar an opportunity to reflect on their performance.	◯ Not for any consultation	◯ Yes, for 1 consultation	◯ Yes, for 2 consultations	◯ Yes, for 3 or more consultations	
	RCA-CTV	RTO1	a. I gave the registrar an opportunity to reflect on their performance.	◯ Not for any case	◯ Yes, for 1 case	◯ Yes, for 2 cases	◯ Yes, for 3 or more cases	
	CBD-CTV		a. I gave the registrar an opportunity to reflect on their performance.	◯ Not for any case	◯ Yes, for 1 case	◯ Yes, for 2 cases	◯ Yes, for 3 or more cases	

	Face-to-face CTV **and** Video/phone	ALL RTOs	b. I gave feedback to the registrar on a specific aspect of their performance	◯ Not for any consultation	◯ Yes, for 1 consultation	◯ Yes, for 2 consultations	◯ Yes, for 3 or more consultations
	RCA-CTV	RTO1	b. I gave feedback to the registrar on a specific aspect of their performance	◯ Not for any case	◯ Yes, for 1 case	◯ Yes, for 2 cases	◯ Yes, for 3 or more cases
	CBD-CTV		b. I gave feedback to the registrar on a specific aspect of their performance	◯ Not for any case	◯ Yes, for 1 case	◯ Yes, for 2 cases	◯ Yes, for 3 or more cases
	Face-to-face CTV **only**	ALL RTOs	c. I <u>observed</u> the registrar performing a physical examination (other than BP and pulse measurement).	◯ Not for any consultation	◯ Yes, for 1 consultation	◯ Yes, for 2 consultations	◯ Yes, for 3 or more consultations
11	Face-to-face CTV **and** Video/phone	ALL RTOs	Regarding the consultations observed, which of the following areas (if any) did you <u>discuss</u> with the registrar? Please select all that apply:	□ Identifying the reason for the consultation<br>□ Exploration of patient's problems<br>□ Consideration of the patient's agenda<br>□ Organisation and flow<br>□ Non-verbal behaviour<br>□ Developing rapport<br>□ Appropriateness of physical examination components performed/proposed<br>□Physical examination technique<br>□ Diagnosis<br>□ Explanation of diagnosis to patient<br>□ Specific patient and/or location contextual factors relevant to the consultation(s)<br>*(e.g. rural/remote, socioeconomic, Aboriginal and/or Torres Strait Islander, non-English speaking background factors)*		□ Time management<br>□ Management planning<br>□ Appropriate medications<br>□ Appropriate investigations<br>□ Medication prescribing processes<br>□ Documentation in patients' medical records<br>□ Referrals<br>□ Patient follow-up<br>□ Dealing with uncertainty<br>□ Safety netting<br>*(Contingency planning with the patient to provide a management strategy for a change in symptoms, including explicit instruction for action(s) given specific circumstances)*	
Note for item 11, response options are the same across all formats. Variation in question terminology only.	RCA-CTV	RTO1 only	Regarding the cases reviewed, which of the following areas (if any) did you <u>discuss</u> with the registrar? Please select all that apply:				
	CBD-CTV		Regarding the cases reviewed, which of the following areas (if any) did you <u>discuss</u> with the registrar? Please select all that apply:				
12	Face-to-face CTV **and** Video/phone	ALL RTOs	Is there anything else you would like to comment on regarding the content, educational usefulness, or any other aspect of the CTV?	________________________________			

	RCA-CTV	RTO1 only	Is there anything else you would like to comment on regarding the content, educational usefulness, or any other aspect of the RCA-CTV?	___________________________
	CBD-CTV		Is there anything else you would like to comment on regarding the content, educational usefulness, or any other aspect of the CBD-CTV?	___________________________
13	All	ALL RTOs	Is this the first time you have participated in this questionnaire (in 2020) about a CTV, RCA-CTV, or CBD-CTV?	○ Yes ○ No ○ Unsure
14	All	ALL RTOs	What best describes your gender?	○ Male ○ Female ○ Prefer to self-describe as: __________ (optional) ○ Prefer not to say
15	All	ALL RTOs	Your age	___________________________
16	All	ALL RTOs	Where did you qualify as a doctor (primary medical degree)?	○ Australia ○ Other, please specify __________________
17	All	ALL RTOs	Which specialist GP Fellowship did you complete? (tick all that are applicable)	□ FRACGP □ FARGP □ ACRRM □ Other (optional please specify): _________
18	All	ALL RTOs	How many years since you gained fellowship?	___________________________________
19	All	ALL RTOs	How many hours do you work each week as a GP?<br>*Include only face-to-face, rostered, patient consultation time*	____________________________________
20	All	ALL RTOs	What best describes the location of your current practice?	○ Major city (RA1) ○ Inner regional area (RA2) ○ Outer regional area (RA3) ○ Remote (RA4) ○ Very remote (RA5)
21	All	ALL RTOs	Are you currently or have you previously (within the past five years) been in any of the following medical education roles?	

			Medical Educator (vocational training)	◯ Currently	◯ Previously	◯ N/A
			Accredited GP supervisor	◯ Currently	◯ Previously	◯ N/A
			Examiner for ACRRM Fellowship exams	◯ Currently	◯ Previously	◯ N/A
			Examiner for RACGP Fellowship exams	◯ Currently	◯ Previously	◯ N/A
			Clinical Teaching Visitor	◯ Currently	◯ Previously	◯ N/A
22	All	ALL RTOs	How many years have you been performing CTVs?	____		
