# Supplementary material for: Educational utility of observational workplace-based assessment modalities in Australian vocational general practice training: a cross-sectional study
Source: BMC Med Educ. 2025 May 23;25:762. doi: 10.1186/s12909-025-07328-y (PMC12102905; doi:10.1186/s12909-025-07328-y)
Supplement: Supplementary file 2 — Supplementary Material 2. [file 12909_2025_7328_MOESM2_ESM.docx]

**Supplementary Table 1. Frequencies and percentages of each outcome measure by ECTV modality**

| **Outcome 1 - Registrar overall perceived educational utility rating** | | | | | | | | | |
| --- | --- | --- | --- | --- | --- | --- | --- | --- | --- |
|  | ECTV modality | | | | | | | | |
| Rating | Face-to-face  n (%) | Video/Phone  n (%) | | CNA  n (%) | | CBD  n (%) | | | Total |
| 1 – Not at all useful | 0 | 3 (0.57) | | 1 (0.97) | | 0 | | | 4 |
| 2 | 3 (2.94) | 4 (0.76) | | 0 | | 0 | | | 7 |
| 3 | 10 (9.80) | 37 (7.07) | | 3 (2.91) | | 1 (7.69) | | | 51 |
| 4 | 31 (30.39) | 150 (28.68) | | 21 (20.39) | | 2 (15.38) | | | 204 |
| 5 – Very useful | 58 (56.86) | 329 (62.91) | | 78 (75.73) | | 10 (76.92) | | | 475 |
| Total | 102 | 523 | | 103 | | 13 | | | 471 |
| **Outcome 2 - registrar rating of likelihood to change practice** | | | | | | | | | |
|  | Likelihood to change practice | | | | | | | | |
| Rating | Face-to-face  n (%) | | Video/Phone  n (%) | | CNA  n (%) | | CBD  n (%) | Total | |
| 1 – Not at all likely | 1 (0.98) | | 1 (0.19) | | 1 (0.98) | | 0 | 3 | |
| 2 | 3 (2.94) | | 14 (2.71) | | 0 | | 0 | 17 | |
| 3 | 17 (16.67) | | 56 (10.83) | | 5 (4.90) | | 0 | 78 | |
| 4 | 31 (30.39) | | 151 (29.21) | | 20 (19.61) | | 5 (38.46) | 207 | |
| 5 – Very likely | 50 (49.02) | | 295 (57.06) | | 76 (74.51) | | 8 (61.54) | 429 | |
| Total | 102 | | 517 | | 102 | | 13 | 734 | |
| **Outcome 3 - registrar’s rating of likelihood to change their learning/training** | | | | | | | | | |
|  | Likelihood to change learning/training | | | | | | | | |
| Rating | Face-to-face  n (%) | | Video/Phone  n (%) | | CNA  n (%) | | CBD  n (%) | Total | |
| 1 – Not at all likely | 1 (0.98) | | 4 (0.77) | | 1 (0.97) | | 0 | 6 | |
| 2 | 7 (6.86) | | 16 (3.09) | | 3 (2.91) | | 2 (15.38) | 28 | |
| 3 | 19 (18.63) | | 83 (16.05) | | 12 (11.65) | | 0 | 114 | |
| 4 | 33 (32.35) | | 173 (33.46) | | 24 (23.30) | | 5 (38.46) | 235 | |
| 5 – Very likely | 42 (41.18) | | 241 (46.62) | | 63 (61.17) | | 6 (46.15) | 352 | |
| Total | 102 | | 517 | | 103 | | 13 | 735 | |
| **Outcome 4 – ECT visitor perceived overall educational utility rating** | | | | | | | | | |
|  | ECTV modality | | | | | | | | |
| Rating | Face-to-face  n (%) | Video/Phone  n (%) | | CNA  n (%) | | CBD  n (%) | | | Total |
| 1 – Not at all useful | 0 | 0 | | 0 | | 0 | | | 0 |
| 2 | 0 | 3 (0.63) | | 2 (1.83) | | 1(5.88) | | | 6 |
| 3 | 7 (7.53) | 67 (14.05) | | 14 (12.84) | | 0 | | | 88 |
| 4 | 50 (53.76) | 258 (54.09) | | 49 (44.95) | | 10 (58.82) | | | 367 |
| 5 – Very useful | 36 (38.71) | 149 (31.24) | | 44 (40.37) | | 6 (35.29) | | | 235 |
| Total | 93 | 477 | | 109 | | 17 | | | 696 |

**Supplementary Table 2. Univariate analysis of registrar educational usefulness rating.**

|  | | **Registrar Perceived Usefulness of CTV** | | |
| --- | --- | --- | --- | --- |
| **Covariate** | **Class** | **Rated 1-4** | **Rated 5** | **p** |
| Registrar pathway | General | 147 (56%) | 269 (57%) | .66 |
|  | Rural | 114 (44%) | 205 (43%) |  |
| Registrar term | GPT 1 | 98 (38%) | 179 (38%) | .96 |
|  | GPT 2 | 112 (43%) | 212 (45%) |  |
|  | GPT 3 | 50 (19%) | 83 (18%) |  |
| Registrar FT/PT | Full-time | 210 (81%) | 385 (81%) | .63 |
|  | Part-time | 50 (19%) | 89 (19%) |  |
| Number of GPs in practice | <2GPs | 25 (10%) | 43 (9%) | .63 |
|  | 2-4 GPs | 105 (40%) | 213 (45%) |  |
|  | 5-9 GPs | 101 (39%) | 165 (35%) |  |
|  | >10 GPs | 29 (11%) | 53 (11%) |  |
| Location of practice | MMM 1 | 137 (52%) | 236 (50%) | .70 |
|  | MMM 2-3 | 71 (27%) | 119 (25%) |  |
|  | MMM 4-7 | 58 (22%) | 120 (25%) |  |
| Quality of feedback | Rated 1-4 | 161 (61%) | 35 (7%) | <.001 |
|  | Rated 5 | 102 (39%) | 438 (93%) |  |
| Consistency of feedback | Rated 1-4 | 133 (51%) | 162 (34%) | <.001 |
|  | Rated 5 | 127 (49%) | 308 (66%) |  |
| Rurality of Visitors practice | Major city | 99 (53%) | 191 (56%) | .83 |
|  | Inner regional | 46 (25%) | 68 (20%) |  |
|  | Outer regional | 41 (22%) | 82 (24%) |  |
| CT Visitor experience as medical educator | Currently | 90 (48%) | 139 (41%) | .45 |
|  | Previously | 34 (18%) | 74 (22%) |  |
|  | N/A | 62 (33%) | 128 (38%) |  |
| CT Visitor experience as GP Supervisor | Currently | 78 (42%) | 122 (36%) | .32 |
|  | Previously | 31 (17%) | 81 (24%) |  |
|  | N/A | 77 (41%) | 138 (40%) |  |
| CT Visitor experience as ACRRM/RACGP examiner | Currently | 73 (39%) | 124 (36%) | .36 |
|  | Previously | 18 (10%) | 47 (14%) |  |
|  | N/A | 95 (51%) | 170 (50%) |  |
| Registrar gender | Female | 152 (61%) | 281 (60%) | .94 |
|  | Male | 97 (39%) | 189 (40%) |  |
| Registrar AMG/IMG | Australia | 201 (77%) | 327 (69%) | .10 |
|  | IMG | 59 (23%) | 147 (31%) |  |
| Type of Assessment | Face-to-face | 44 (17%) | 58 (12%) | .12 |
|  | Video/phone | 194 (73%) | 329 (69%) |  |
|  | CNA CTV | 25 (9%) | 78 (16%) |  |
|  | CBD CTV | 3 (1%) | 10 (2%) |  |
| Registrar able to reflect on performance | 0-2 cases | 27 (10%) | 36 (8%) | .21 |
|  | 3 or more cases | 239 (90%) | 439 (92%) |  |
| Registrar provided with meaningful feedback | 0-2 cases | 44 (17%) | 13 (3%) | <.001 |
|  | 3 or more cases | 222 (83%) | 462 (97%) |  |
| RTO | 1 | 240 (90%) | 443 (93%) | .005 |
|  | 2 | 20 (8%) | 7 (1%) |  |
|  | 3 | 6 (2%) | 25 (5%) |  |
| Discussed consideration of patient’s agenda | No | 116 (44%) | 170 (36%) | .02 |
|  | Yes | 147 (56%) | 303 (64%) |  |
| Discussed organisation and flow | No | 145 (55%) | 218 (46%) | .09 |
|  | Yes | 118 (45%) | 255 (54%) |  |
| Discussed developing rapport | No | 149 (57%) | 225 (48%) | .03 |
|  | Yes | 114 (43%) | 248 (52%) |  |
| Discussed specific contextual factors | No | 217 (83%) | 312 (66%) | <.001 |
|  | Yes | 46 (17%) | 161 (34%) |  |
| Discussed time management | No | 154 (59%) | 251 (53%) | .34 |
|  | Yes | 109 (41%) | 222 (47%) |  |
| Discussed management planning | No | 161 (61%) | 188 (40%) | <.001 |
|  | Yes | 102 (39%) | 285 (60%) |  |
| Discussed appropriate medications | No | 188 (71%) | 258 (55%) | .002 |
|  | Yes | 75 (29%) | 215 (45%) |  |
| Discussed appropriate investigations | No | 145 (55%) | 198 (42%) | .007 |
|  | Yes | 118 (45%) | 275 (58%) |  |
| Discussed patient follow-up | No | 133 (51%) | 166 (35%) | .001 |
|  | Yes | 130 (49%) | 307 (65%) |  |
| Discussed dealing with uncertainty | No | 159 (60%) | 214 (45%) | <.001 |
|  | Yes | 104 (40%) | 259 (55%) |  |
| Discussed physical examination | No | 158 (60%) | 223 (47%) | .004 |
|  | Yes | 105 (40%) | 250 (53%) |  |
| CT Visitor gender | Female | 121 (66%) | 240 (72%) | .23 |
|  | Male | 62 (34%) | 95 (28%) |  |
| CT Visitor AMG/IMG | Australia | 157 (84%) | 287 (84%) | .85 |
|  | IMG | 29 (16%) | 54 (16%) |  |
| SEIFA-IRSD | mean (SD) | 5.70 (2.87) | 5.60 (2.92) | .52 |
| CT Visitor years of experience | mean (SD) | 6.83 (6.48) | 6.45 (6.28) | .49 |
| CT Visitor years since gaining fellowship | mean (SD) | 13.02 (10.74) | 12.21 (9.57) | .41 |
| Registrar age | mean (SD) | 33.45 (6.35) | 34.06 (6.73) | .57 |
| Number of cases | mean (SD) | 4.96 (1.48) | 4.77 (1.38) | .10 |
| CT Visitor age | mean (SD) | 47.07 (12.69) | 46.85 (11.76) | .58 |
| CT Visitor weekly hours | mean (SD) | 21.25 (10.78) | 20.94 (10.17) | .78 |

# Supplementary Table 3. Univariate analysis of registrar perceived likelihood of changing practice.

|  | | **Registrar Likelihood of changing practice** | | |
| --- | --- | --- | --- | --- |
| **Covariate** | **Class** | **Rated 1-4** | **Rated 5** | **p** |
| Registrar pathway | General | 169 (56%) | 246 (57%) | .66 |
|  | Rural | 133 (44%) | 183 (43%) |  |
| Registrar term | GPT 1 | 115 (38%) | 161 (38%) | .56 |
|  | GPT 2 | 127 (42%) | 195 (45%) |  |
|  | GPT 3 | 59 (20%) | 73 (17%) |  |
| Registrar FT/PT | Full-time | 244 (81%) | 348 (81%) | .79 |
|  | Part-time | 57 (19%) | 81 (19%) |  |
| Number of GPs in practice | <2GPs | 23 (8%) | 45 (10%) | .37 |
|  | 2-4 GPs | 133 (44%) | 183 (43%) |  |
|  | 5-9 GPs | 107 (36%) | 157 (37%) |  |
|  | >10 GPs | 38 (13%) | 44 (10%) |  |
| Location of practice | MMM 1 | 150 (49%) | 219 (51%) | .66 |
|  | MMM 2-3 | 80 (26%) | 108 (25%) |  |
|  | MMM 4-7 | 75 (25%) | 102 (24%) |  |
| Quality of feedback | Rated 1-4 | 131 (43%) | 65 (15%) | <.001 |
|  | Rated 5 | 174 (57%) | 364 (85%) |  |
| Consistency of feedback | Rated 1-4 | 156 (52%) | 138 (32%) | <.001 |
|  | Rated 5 | 146 (48%) | 289 (68%) |  |
| Rurality of Visitors practice | Major city | 116 (51%) | 172 (58%) | .42 |
|  | Inner regional | 56 (25%) | 58 (20%) |  |
|  | Outer regional | 55 (24%) | 66 (22%) |  |
| CT Visitor experience as medical educator | Currently | 98 (43%) | 130 (44%) | .90 |
|  | Previously | 46 (20%) | 60 (20%) |  |
|  | N/A | 83 (37%) | 106 (36%) |  |
| CT Visitor experience as GP Supervisor | Currently | 86 (38%) | 113 (38%) | .69 |
|  | Previously | 44 (19%) | 67 (23%) |  |
|  | N/A | 97 (43%) | 116 (39%) |  |
| CT Visitor experience as ACRRM/RACGP examiner | Currently | 77 (34%) | 119 (40%) | .17 |
|  | Previously | 24 (11%) | 41 (14%) |  |
|  | N/A | 126 (56%) | 136 (46%) |  |
| Registrar gender | Female | 173 (60%) | 257 (60%) | .72 |
|  | Male | 115 (40%) | 170 (40%) |  |
| Registrar AMG/IMG | Australia | 240 (80%) | 285 (66%) | .002 |
|  | IMG | 61 (20%) | 144 (34%) |  |
| Type of Assessment | Face-to-face | 52 (17%) | 50 (12%) | .009 |
|  | Video/phone | 222 (73%) | 295 (69%) |  |
|  | CNA CTV | 26 (9%) | 76 (18%) |  |
|  | CBD CTV | 5 (2%) | 8 (2%) |  |
| Registrar able to reflect on performance | 0-2 cases | 35 (11%) | 27 (6%) | .009 |
|  | 3 or more cases | 270 (89%) | 402 (94%) |  |
| Registrar provided with meaningful feedback | 0-2 cases | 39 (13%) | 17 (4%) | <.001 |
|  | 3 or more cases | 266 (87%) | 412 (96%) |  |
| RTO | 1 | 273 (90%) | 403 (94%) | .05 |
|  | 2 | 20 (7%) | 7 (2%) |  |
|  | 3 | 12 (4%) | 19 (4%) |  |
| Discussed consideration of patient’s agenda | No | 132 (43%) | 153 (36%) | .009 |
|  | Yes | 173 (57%) | 276 (64%) |  |
| Discussed organisation and flow | No | 164 (54%) | 198 (46%) | .04 |
|  | Yes | 141 (46%) | 231 (54%) |  |
| Discussed developing rapport | No | 163 (53%) | 210 (49%) | .15 |
|  | Yes | 142 (47%) | 219 (51%) |  |
| Discussed specific contextual factors | No | 231 (76%) | 297 (69%) | .03 |
|  | Yes | 74 (24%) | 132 (31%) |  |
| Discussed time management | No | 182 (60%) | 222 (52%) | .006 |
|  | Yes | 123 (40%) | 207 (48%) |  |
| Discussed management planning | No | 171 (56%) | 177 (41%) | <.001 |
|  | Yes | 134 (44%) | 252 (59%) |  |
| Discussed appropriate medications | No | 209 (69%) | 236 (55%) | <.001 |
|  | Yes | 96 (31%) | 193 (45%) |  |
| Discussed appropriate investigations | No | 165 (54%) | 177 (41%) | .001 |
|  | Yes | 140 (46%) | 252 (59%) |  |
| Discussed patient follow-up | No | 134 (44%) | 165 (38%) | .07 |
|  | Yes | 171 (56%) | 264 (62%) |  |
| Discussed dealing with uncertainty | No | 166 (54%) | 206 (48%) | .04 |
|  | Yes | 139 (46%) | 223 (52%) |  |
| Discussed physical examination | No | 170 (56%) | 210 (49%) | .06 |
|  | Yes | 135 (44%) | 219 (51%) |  |
| CT Visitor gender | Female | 148 (67%) | 211 (72%) | .13 |
|  | Male | 74 (33%) | 81 (28%) |  |
| CT Visitor AMG/IMG | Australia | 196 (86%) | 247 (83%) | .31 |
|  | IMG | 31 (14%) | 49 (17%) |  |
| SEIFA-IRSD | mean (SD) | 5.72 (2.81) | 5.59 (2.96) | .79 |
| CT Visitor years of experience | mean (SD) | 6.45 (6.34) | 6.68 (6.38) | .76 |
| CT Visitor years since gaining fellowship | mean (SD) | 11.94 (9.91) | 12.79 (9.88) | .34 |
| Registrar age | mean (SD) | 33.41 (6.45) | 34.14 (6.71) | .37 |
| Number of cases | mean (SD) | 4.85 (1.46) | 4.84 (1.39) | .87 |
| CT Visitor age | mean (SD) | 46.59 (12.46) | 47.05 (11.72) | .80 |
| CT Visitor weekly hours | mean (SD) | 20.77 (10.65) | 21.32 (10.16) | .44 |

# Supplementary Table 4. Univariate analysis of registrar perceived likelihood of changing approach to learning/training.

|  | | **Registrar Likelihood of changing approach to learning** | | |
| --- | --- | --- | --- | --- |
| **Covariate** | **Class** | **Rated 1-4** | **Rated 5** | **p** |
| Registrar pathway | General | 223 (59%) | 192 (55%) | .29 |
|  | Rural | 157 (41%) | 160 (45%) |  |
| Registrar term | GPT 1 | 140 (37%) | 135 (38%) | .12 |
|  | GPT 2 | 159 (42%) | 164 (47%) |  |
|  | GPT 3 | 80 (21%) | 53 (15%) |  |
| Registrar FT/PT | Full-time | 307 (81%) | 285 (81%) | .63 |
|  | Part-time | 72 (19%) | 67 (19%) |  |
| Number of GPs in practice | <2GPs | 33 (9%) | 35 (10%) | .70 |
|  | 2-4 GPs | 161 (42%) | 155 (44%) |  |
|  | 5-9 GPs | 140 (37%) | 125 (36%) |  |
|  | >10 GPs | 45 (12%) | 37 (11%) |  |
| Location of practice | MMM 1 | 196 (51%) | 173 (49%) | .74 |
|  | MMM 2-3 | 100 (26%) | 89 (25%) |  |
|  | MMM 4-7 | 87 (23%) | 90 (26%) |  |
| Consistency of feedback | Rated 1-4 | 194 (51%) | 101 (29%) | <.001 |
|  | Rated 5 | 187 (49%) | 247 (71%) |  |
| Rurality of Visitors practice | Major city | 149 (53%) | 139 (57%) | .53 |
|  | Inner regional | 61 (22%) | 52 (21%) |  |
|  | Outer regional | 69 (25%) | 53 (22%) |  |
| CT Visitor experience as medical educator | Currently | 122 (44%) | 106 (43%) | .97 |
|  | Previously | 58 (21%) | 48 (20%) |  |
|  | N/A | 99 (35%) | 90 (37%) |  |
| CT Visitor experience as GP Supervisor | Currently | 110 (39%) | 89 (36%) | .42 |
|  | Previously | 53 (19%) | 58 (24%) |  |
|  | N/A | 116 (42%) | 97 (40%) |  |
| CT Visitor experience as ACRRM/RACGP examiner | Currently | 99 (35%) | 98 (40%) | .80 |
|  | Previously | 32 (11%) | 32 (13%) |  |
|  | N/A | 148 (53%) | 114 (47%) |  |
| Registrar gender | Female | 220 (60%) | 211 (60%) | .61 |
|  | Male | 147 (40%) | 138 (40%) |  |
| Registrar AMG/IMG | Australia | 310 (82%) | 215 (61%) | <.001 |
|  | IMG | 69 (18%) | 137 (39%) |  |
| Type of Assessment | Face-to-face | 60 (16%) | 42 (12%) | .04 |
|  | Video/phone | 276 (72%) | 241 (68%) |  |
|  | CNA CTV | 40 (10%) | 63 (18%) |  |
|  | CBD CTV | 7 (2%) | 6 (2%) |  |
| Registrar able to reflect on performance | 0-2 cases | 34 (9%) | 28 (8%) | .63 |
|  | 3 or more cases | 349 (91%) | 324 (92%) |  |
| Registrar provided with meaningful feedback | 0-2 cases | 47 (12%) | 9 (3%) | <.001 |
|  | 3 or more cases | 336 (88%) | 343 (97%) |  |
| RTO | 1 | 347 (91%) | 330 (94%) | .05 |
|  | 2 | 23 (6%) | 4 (1%) |  |
|  | 3 | 13 (3%) | 18 (5%) |  |
| Discussed consideration of patient’s agenda | No | 158 (41%) | 127 (36%) | .07 |
|  | Yes | 225 (59%) | 225 (64%) |  |
| Discussed organisation and flow | No | 200 (52%) | 163 (46%) | .29 |
|  | Yes | 183 (48%) | 189 (54%) |  |
| Discussed developing rapport | No | 205 (54%) | 169 (48%) | .12 |
|  | Yes | 178 (46%) | 183 (52%) |  |
| Discussed specific contextual factors | No | 297 (78%) | 231 (66%) | <.001 |
|  | Yes | 86 (22%) | 121 (34%) |  |
| Discussed time management | No | 225 (59%) | 180 (51%) | .02 |
|  | Yes | 158 (41%) | 172 (49%) |  |
| Discussed management planning | No | 215 (56%) | 133 (38%) | <.001 |
|  | Yes | 168 (44%) | 219 (62%) |  |
| Discussed appropriate medications | No | 261 (68%) | 184 (52%) | <.001 |
|  | Yes | 122 (32%) | 168 (48%) |  |
| Discussed appropriate investigations | No | 204 (53%) | 138 (39%) | <.001 |
|  | Yes | 179 (47%) | 214 (61%) |  |
| Discussed patient follow-up | No | 161 (42%) | 137 (39%) | .22 |
|  | Yes | 222 (58%) | 215 (61%) |  |
| Discussed dealing with uncertainty | No | 216 (56%) | 156 (44%) | <.001 |
|  | Yes | 167 (44%) | 196 (56%) |  |
| Discussed physical examination | No | 216 (56%) | 164 (47%) | .005 |
|  | Yes | 167 (44%) | 188 (53%) |  |
| CT Visitor gender | Female | 190 (69%) | 170 (71%) | .78 |
|  | Male | 84 (31%) | 70 (29%) |  |
| CT Visitor AMG/IMG | Australia | 239 (86%) | 203 (83%) | .18 |
|  | IMG | 40 (14%) | 41 (17%) |  |
| SEIFA-IRSD | mean (SD) | 5.81 (2.83) | 5.45 (2.98) | .09 |
| CT Visitor years of experience | mean (SD) | 6.71 (6.42) | 6.44 (6.29) | .42 |
| CT Visitor years since gaining fellowship | mean (SD) | 12.50 (9.97) | 12.30 (9.83) | .41 |
| Registrar age | mean (SD) | 33.12 (6.40) | 34.61 (6.72) | .02 |
| Number of cases | mean (SD) | 4.83 (1.39) | 4.87 (1.45) | .73 |
| CT Visitor age | mean (SD) | 47.09 (12.36) | 46.59 (11.69) | .32 |
| CT Visitor weekly hours | mean (SD) | 20.65 (10.67) | 21.69 (9.93) | .16 |

# Supplementary Table 5. Univariate analysis of CT visitor perceived overall educational usefulness rating.

|  | | **Visitor Perceived usefulness of CTV** | | |
| --- | --- | --- | --- | --- |
| **Covariate** | **Class** | **Rated 1-4** | **Rated 5** | **p** |
| Registrar pathway | General | 138 (54%) | 85 (64%) | .32 |
|  | Rural | 119 (46%) | 48 (36%) |  |
| Registrar term | GPT 1 | 128 (50%) | 72 (54%) | .53 |
|  | GPT 2 | 89 (35%) | 38 (29%) |  |
|  | GPT 3 | 39 (15%) | 23 (17%) |  |
| Registrar FT/PT | Full-time | 204 (80%) | 107 (80%) | .67 |
|  | Part-time | 52 (20%) | 26 (20%) |  |
| Number of GPs in practice | <2GPs | 25 (10%) | 13 (10%) | .93 |
|  | 2-4 GPs | 115 (45%) | 63 (47%) |  |
|  | 5-9 GPs | 83 (32%) | 40 (30%) |  |
|  | >10 GPs | 33 (13%) | 17 (13%) |  |
| Location of practice | MMM 1 | 239 (52%) | 144 (61%) | .24 |
|  | MMM 2-3 | 113 (25%) | 43 (18%) |  |
|  | MMM 4-7 | 109 (24%) | 48 (20%) |  |
| Rurality of Visitors practice | Major city | 240 (55%) | 145 (64%) | .14 |
|  | Inner regional | 99 (23%) | 35 (15%) |  |
|  | Outer regional | 100 (23%) | 47 (21%) |  |
| CT Visitor experience as medical educator | Currently | 183 (42%) | 103 (45%) | .19 |
|  | Previously | 104 (24%) | 28 (12%) |  |
|  | N/A | 152 (35%) | 96 (42%) |  |
| CT Visitor experience as GP Supervisor | Currently | 179 (41%) | 87 (38%) | .07 |
|  | Previously | 64 (15%) | 62 (27%) |  |
|  | N/A | 196 (45%) | 78 (34%) |  |
| CT Visitor experience as ACRRM/RACGP examiner | Currently | 172 (39%) | 100 (44%) | .19 |
|  | Previously | 57 (13%) | 27 (12%) |  |
|  | N/A | 210 (48%) | 100 (44%) |  |
| Registrar gender | Female | 148 (59%) | 77 (58%) | .81 |
|  | Male | 102 (41%) | 55 (42%) |  |
| Registrar AMG/IMG | Australia | 189 (74%) | 101 (76%) | .90 |
|  | IMG | 67 (26%) | 32 (24%) |  |
| CT Visitor gender | Female | 335 (77%) | 141 (63%) | .04 |
|  | Male | 98 (23%) | 82 (37%) |  |
| CT Visitor AMG/IMG | Australia | 362 (82%) | 189 (83%) | .62 |
|  | IMG | 77 (18%) | 38 (17%) |  |
| Type of Assessment | Face-to-face | 57 (12%) | 36 (15%) | .10 |
|  | Video/phone | 328 (71%) | 149 (63%) |  |
|  | CNA CTV | 65 (14%) | 44 (19%) |  |
|  | CBD CTV | 11 (2%) | 6 (3%) |  |
| Registrar able to reflect on performance | 0-2 cases | 100 (22%) | 21 (9%) | .002 |
|  | 3 or more cases | 361 (78%) | 214 (91%) |  |
| Registrar provided with meaningful feedback | 0-2 cases | 49 (11%) | 8 (3%) | .03 |
|  | 3 or more cases | 412 (89%) | 227 (97%) |  |
| RTO | 1 | 411 (89%) | 213 (91%) | .10 |
|  | 2 | 33 (7%) | 10 (4%) |  |
|  | 3 | 17 (4%) | 12 (5%) |  |
| Discussed consideration of patient’s agenda | No | 160 (35%) | 61 (26%) | .04 |
|  | Yes | 301 (65%) | 174 (74%) |  |
| Discussed organisation and flow | No | 230 (50%) | 91 (39%) | .004 |
|  | Yes | 231 (50%) | 144 (61%) |  |
| Discussed developing rapport | No | 255 (55%) | 126 (54%) | .92 |
|  | Yes | 206 (45%) | 109 (46%) |  |
| Discussed specific contextual factors | No | 315 (68%) | 156 (66%) | .89 |
|  | Yes | 146 (32%) | 79 (34%) |  |
| Discussed time management | No | 210 (46%) | 113 (48%) | .71 |
|  | Yes | 251 (54%) | 122 (52%) |  |
| Discussed management planning | No | 191 (41%) | 71 (30%) | .003 |
|  | Yes | 270 (59%) | 164 (70%) |  |
| Discussed appropriate medications | No | 272 (59%) | 124 (53%) | .08 |
|  | Yes | 189 (41%) | 111 (47%) |  |
| Discussed appropriate investigations | No | 219 (48%) | 99 (42%) | .06 |
|  | Yes | 242 (52%) | 136 (58%) |  |
| Discussed patient follow-up | No | 157 (34%) | 71 (30%) | .07 |
|  | Yes | 304 (66%) | 164 (70%) |  |
| Discussed dealing with uncertainty | No | 265 (57%) | 123 (52%) | .25 |
|  | Yes | 196 (43%) | 112 (48%) |  |
| Discussed physical examination | No | 265 (57%) | 111 (47%) | .02 |
|  | Yes | 196 (43%) | 124 (53%) |  |
| SEIFA-IRSD | mean (SD) | 5.45 (2.84) | 6.10 (2.95) | .11 |
| CT Visitor years of experience | mean (SD) | 6.74 (6.22) | 7.55 (7.26) | .52 |
| CT Visitor years since gaining fellowship | mean (SD) | 13.11 (10.52) | 13.19 (9.94) | .83 |
| Registrar age | mean (SD) | 33.81 (6.26) | 33.29 (6.48) | .85 |
| Registrar weekly hours | mean (SD) | 34.88 (6.45) | 34.86 (7.00) | .67 |
| CT Visitor age | mean (SD) | 47.02 (12.08) | 48.41 (12.01) | .33 |
| CT Visitor weekly hours | mean (SD) | 20.46 (10.23) | 20.73 (11.64) | .58 |
| Number of cases | mean (SD) | 4.65 (1.23) | 4.66 (1.18) | .57 |

# Supplementary Table 6. Comparison of Full and Multiple Imputation (MI) multivariable models including all covariates of interest for registrar educational usefulness rating.

|  |  | **Full model** | | **MI model** | |
| --- | --- | --- | --- | --- | --- |
| **Covariate** | **Class** | **OR (95% CI)** | **p-value** | **OR (95% CI)** | **p-value** |
| Type of assessment | Video/phone | 0.60 (0.27, 1.31) | .20 | 0.56 (0.25, 1.28) | .17 |
|  | CNA CTV | 0.90 (0.30, 2.76) | .86 | 0.90 (0.28, 2.95) | .87 |
|  | CBD CTV | 0.85 (0.06, 13.2) | .91 | 0.89 (0.04, 19.14) | .94 |
| Registrar gender | Female | 0.74 (0.42, 1.28) | .28 | 0.71 (0.40, 1.27) | .25 |
| Registrar Age |  | 0.96 (0.92, 1.00) | .06 | 0.96 (0.92, 1.01) | .11 |
| Registrar AMG/IMG | IMG | 1.54 (0.76, 3.11) | .23 | 1.54 (0.74, 3.17) | .25 |
| Registrar training term | GPT 2 | 0.91 (0.56, 1.49) | .72 | 0.91 (0.54, 1.54) | .72 |
|  | GPT 3 | 0.56 (0.27, 1.17) | .13 | 0.53 (0.24, 1.14) | .10 |
| Registrar FT/PT | Part time | 1.34 (0.68, 2.61) | .40 | 1.28 (0.64, 2.55) | .48 |
| Registrar pathway | Rural | 1.00 (0.35, 2.89) | .99 | 0.89 (0.29, 2.74) | .84 |
| SEIFA-IRSD |  | 0.97 (0.88, 1.05) | .43 | 0.97 (0.89, 1.07) | .57 |
| Location of practice | MMM 2-3 | 0.81 (0.31, 2.09) | .66 | 0.90 (0.32, 2.47) | .83 |
|  | MMM 4-7 | 1.07 (0.37, 3.05) | .90 | 1.10 (0.36, 3.37) | .86 |
| Number of GPs in practice | 2-4 GPs | 1.89 (0.90, 4.00) | .09 | 1.93 (0.90, 4.18) | .09 |
|  | 5-9 GPs | 1.13 (0.52, 2.46) | .77 | 1.11 (0.50, 2.48) | .80 |
|  | >10 GPs | 1.17 (0.46, 2.96) | .75 | 1.17 (0.44, 3.08) | .75 |
| Number of cases |  | 1.01 (0.85, 1.21) | .90 | 1.00 (0.83, 1.20) | .99 |
| Registrar able to reflect on performance | 3 or more cases | 0.36 (0.12, 1.11) | .08 | 0.35 (0.12, 1.07) | .07 |
| Registrar provided with meaningful feedback | 3 or more cases | 5.58 (1.55, 20.1) | .009 | 5.79 (1.46, 22.91) | .01 |
| CT visitor gender | Female | 0.96 (0.54, 1.70) | .89 | 1.00 (0.54, 1.86) | .999 |
| CT visitor Age |  | 0.97 (0.94, 1.01) | .18 | 0.98 (0.94, 1.02) | .25 |
| CT visitor AMG/IMG | IMG | 1.12 (0.54, 2.31) | .76 | 1.12 (0.51, 2.47) | .78 |
| CT Visitor weekly hours |  | 0.99 (0.96, 1.01) | .33 | 0.99 (0.96, 1.02) | .33 |
| CT Visitor years since gaining fellowship |  | 1.03 (0.98, 1.07) | .24 | 1.03 (0.98, 1.08) | .30 |
| CT Visitor years of experience |  | 1.01 (0.95, 1.07) | .79 | 1.00 (0.94, 1.07) | .91 |
| Rurality of Visitor’s practice | RA 2 | 0.78 (0.40, 1.53) | .48 | 0.75 (0.37, 1.50) | .42 |
|  | RA 3-5 | 1.01 (0.46, 2.20) | .98 | 1.08 (0.47, 2.48) | .86 |
| CT Visitor experience as a medical educator | Currently | 0.54 (0.30, 0.98) | .04 | 0.51 (0.27, 0.97) | .04 |
|  | Previously | 0.85 (0.43, 1.68) | .64 | 0.83 (0.40, 1.73) | .62 |
| CT Visitor experience as a GP supervisor | Currently | 1.08 (0.54, 2.16) | .82 | 1.05 (0.51, 2.17) | .90 |
|  | Previously | 1.20 (0.62, 2.32) | .60 | 1.30 (0.63, 2.67) | .47 |
| CT Visitor experience as ACRRM/RACGP examiner | Currently | 0.69 (0.39, 1.21) | .20 | 0.69 (0.38, 1.24) | .22 |
|  | Previously | 2.57 (1.15, 5.77) | .02 | 2.77 (1.14, 6.72) | .03 |
| Consistency of feedback received | Rated 5 | 1.50 (0.95, 2.38) | .08 | 1.58 (0.98, 2.54) | .06 |
| Quality of feedback received | Rated 5 | 16.1 (8.32, 31.1) | <.001 | 19.26 (9.85, 37.66) | <.001 |
| Discussed patient agenda | Yes | 0.67 (0.39, 1.14) | .14 | 0.66 (0.38, 1.15) | .14 |
| Discussed organisation and flow | Yes | 1.08 (0.66, 1.78) | .75 | 1.11 (0.65, 1.89) | .71 |
| Discussed developing rapport | Yes | 0.91 (0.53, 1.55) | .72 | 0.86 (0.49, 1.50) | .60 |
| Discussed contextual factors | Yes | 1.59 (0.87, 2.91) | .13 | 1.56 (0.82, 2.95) | .17 |
| Discussed time management | Yes | 1.08 (0.65, 1.79) | .77 | 1.11 (0.64, 1.94) | .70 |
| Discussed management planning | Yes | 0.94 (0.57, 1.56) | .82 | 1.00 (0.59, 1.70) | .99 |
| Discussed appropriate medications | Yes | 1.32 (0.68, 2.54) | .41 | 1.34 (0.68, 2.67) | .40 |
| Discussed appropriate investigations | Yes | 1.31 (0.79, 2.15) | .29 | 1.32 (0.78, 2.24) | .301 |
| Discussed patient follow-up | Yes | 1.00 (0.55, 1.84) | .99 | 0.96 (0.51, 1.83) | .91 |
| Discussed dealing with uncertainty | Yes | 1.60 (0.97, 2.63) | .07 | 1.57 (0.93, 2.64) | .09 |
| Discussed physical examination | Yes | 0.81 (0.49, 1.33) | .41 | 0.81 (0.47, 1.40) | .45 |

# Supplementary Table 7. Comparison of Full and Multiple Imputation (MI) multivariable models including all covariates of interest for registrar likelihood to change practice.

|  |  | **Full models** | | **MI models** | |
| --- | --- | --- | --- | --- | --- |
| **Covariate** | **Class** | **OR (95% CI)** | **p-value** | **OR (95% CI)** | **p-value** |
| Type of assessment | Video/phone | 0.79 (0.29, 2.10) | .63 | 0.98 (0.40, 2.37) | .96 |
|  | CNA CTV | 0.95 (0.29, 3.07) | .93 | 1.37 (0.46, 4.09) | .58 |
|  | CBD CTV | 0.51 (0.07, 3.52) | .49 | 0.78 (0.12, 4.93) | .79 |
| Registrar gender | Female | 1.01 (0.60, 1.69) | .97 | 0.96 (0.57, 1.63) | .88 |
| Registrar Age |  | 0.97 (0.93, 1.01) | .20 | 0.98 (0.94, 1.02) | .38 |
| Registrar AMG/IMG | IMG | 2.87 (1.49, 5.52) | .002 | 2.75 (1.41, 5.39) | .003 |
| Registrar training term | GPT 2 | 1.13 (0.71, 1.79) | .60 | 1.05 (0.66, 1.67) | .85 |
|  | GPT 3 | 0.61 (0.32, 1.14) | .12 | 0.55 (0.29, 1.05) | .07 |
| Registrar FT/PT | Part time | 0.96 (0.52, 1.77) | .89 | 0.82 (0.44, 1.51) | .52 |
| Registrar pathway | Rural | 0.63 (0.26, 1.52) | .31 | 0.60 (0.25, 1.42) | .25 |
| RTO | 2 | 0.29 (0.08, 1.07) | .06 | 0.47 (0.14, 1.55) | .21 |
|  | 3 | 1.67 (0.17, 16.5) | .66 | 1.76 (0.18, 17.44) | .63 |
| SEIFA-IRSD |  | 0.95 (0.88, 1.03) | .24 | 0.95 (0.87, 1.03) | .19 |
| Location of practice | MMM 2-3 | 1.13 (0.48, 2.66) | .78 | 1.24 (0.53, 2.89) | .62 |
|  | MMM 4-7 | 1.01 (0.41, 2.52) | .98 | 1.03 (0.41, 2.56) | .95 |
| Number of GPs in practice | 2-4 GPs | 0.67 (0.31, 1.47) | .32 | 0.72 (0.30, 1.71) | .46 |
|  | 5-9 GPs | 0.62 (0.27, 1.45) | .27 | 0.69 (0.28, 1.73) | .43 |
|  | >10 GPs | 0.49 (0.19, 1.25) | .14 | 0.48 (0.17, 1.32) | .16 |
| Number of cases |  | 1.02 (0.86, 1.21) | .81 | 1.00 (0.84, 1.19) | .98 |
| Registrar able to reflect on performance | 3 or more cases | 1.09 (0.40, 2.97) | .86 | 0.89 (0.33, 2.36) | .81 |
| Registrar provided with meaningful feedback | 3 or more cases | 3.32 (1.23, 8.93) | .02 | 3.74 (1.44, 9.71) | .007 |
| CT visitor gender | Female | 1.62 (0.99, 2.67) | .06 | 1.58 (0.95, 2.65) | .08 |
| CT visitor Age |  | 0.98 (0.94, 1.03) | .46 | 0.99 (0.95, 1.03) | .62 |
| CT visitor AMG/IMG | IMG | 1.61 (0.79, 3.30) | .19 | 1.45 (0.72, 2.94) | .30 |
| CT Visitor weekly hours |  | 1.01 (0.99, 1.04) | .25 | 1.01 (0.99, 1.04) | .33 |
| CT Visitor years since gaining fellowship |  | 1.04 (0.99, 1.09) | .08 | 1.04 (0.99, 1.09) | .12 |
| CT Visitor years of experience |  | 1.01 (0.96, 1.07) | .69 | 1.00 (0.94, 1.06) | .97 |
| Rurality of Visitor’s practice | RA 2 | 0.71 (0.40, 1.27) | .25 | 0.66 (0.37, 1.19) | .17 |
|  | RA 3-5 | 0.68 (0.38, 1.22) | .20 | 0.68 (0.36, 1.28) | .23 |
| CT Visitor experience as a medical educator | Currently | 1.65 (0.97, 2.82) | .07 | 1.64 (0.93, 2.90) | .09 |
|  | Previously | 0.85 (0.44, 1.63) | .62 | 0.91 (0.46, 1.79) | .79 |
| CT Visitor experience as a GP supervisor | Currently | 1.23 (0.68, 2.22) | .49 | 1.27 (0.68, 2.35) | .46 |
|  | Previously | 1.55 (0.82, 2.95) | .18 | 1.77 (0.89, 3.51) | .10 |
| CT Visitor experience as ACRRM/RACGP examiner | Currently | 1.08 (0.68, 1.72) | .75 | 1.20 (0.72, 2.00) | .48 |
|  | Previously | 1.46 (0.70, 3.03) | .32 | 1.69 (0.75, 3.83) | .21 |
| Consistency of feedback received | Rated 5 | 1.93 (1.23, 3.03) | .004 | 1.94 (1.23, 3.05) | .004 |
| Quality of feedback received | Rated 5 | 2.68 (1.67, 4.30) | <.001 | 2.91 (1.75, 4.83) | <.001 |
| Discussed patient agenda | Yes | 1.21 (0.72, 2.02) | .47 | 1.05 (0.62, 1.78) | .85 |
| Discussed organisation and flow | Yes | 0.98 (0.63, 1.54) | .94 | 1.08 (0.68, 1.70) | .74 |
| Discussed developing rapport | Yes | 0.83 (0.54, 1.30) | .42 | 0.82 (0.51, 1.31) | .40 |
| Discussed contextual factors | Yes | 1.33 (0.76, 2.35) | .32 | 1.21 (0.68, 2.13) | .52 |
| Discussed time management | Yes | 1.56 (1.02, 2.40) | .04 | 1.55 (0.99, 2.42) | .06 |
| Discussed management planning | Yes | 0.98 (0.63, 1.53) | .93 | 1.03 (0.66, 1.59) | .91 |
| Discussed appropriate medications | Yes | 1.18 (0.70, 1.98) | .54 | 1.16 (0.68, 1.97) | .58 |
| Discussed appropriate investigations | Yes | 1.52 (0.97, 2.37) | .07 | 1.53 (0.97, 2.44) | .07 |
| Discussed patient follow-up | Yes | 0.90 (0.57, 1.43) | .67 | 0.85 (0.52, 1.39) | .53 |
| Discussed dealing with uncertainty | Yes | 0.97 (0.62, 1.52) | .90 | 0.95 (0.60, 1.51) | .83 |
| Discussed physical examination | Yes | 0.97 (0.62, 1.53) | .91 | 0.84 (0.53, 1.34) | .47 |

# Supplementary Table 8. Comparison of Full and Multiple Imputation (MI) multivariable models including all covariates of interest for registrar likelihood to change learning/training.

|  |  | **Full models** | | **MI models** | |
| --- | --- | --- | --- | --- | --- |
| **Covariate** | **Class** | **OR (95% CI)** | **p-value** | **OR (95% CI)** | **p-value** |
| Type of assessment | Video/phone | 1.23 (0.46, 3.34) | .68 | 0.99 (0.38, 2.57) | .99 |
|  | CNA CTV | 1.65 (0.52, 5.24) | .40 | 1.49 (0.49, 4.57) | .48 |
|  | CBD CTV | 0.30 (0.02, 3.88) | .36 | 0.43 (0.05, 3.79) | .45 |
| Registrar gender | Female | 0.94 (0.55, 1.63) | .83 | 0.86 (0.50, 1.51) | .61 |
| Registrar Age |  | 0.99 (0.95, 1.04) | .75 | 1.00 (0.96, 1.05) | .99 |
| Registrar AMG/IMG | IMG | 3.33 (1.74, 6.36) | <.001 | 3.46 (1.75, 6.85) | <.001 |
| Registrar training term | GPT 2 | 0.97 (0.64, 1.48) | .89 | 1.02 (0.65, 1.59) | .94 |
|  | GPT 3 | 0.27 (0.14, 0.55) | <.001 | 0.27 (0.13, 0.56) | <.001 |
| Registrar FT/PT | Part time | 1.24 (0.68, 2.29) | .48 | 1.02 (0.55, 1.91) | .95 |
| Registrar pathway | Rural | 0.98 (0.41, 2.32) | .96 | 0.83 (0.34, 2.00) | .68 |
| RTO | 2 | 0.29 (0.07, 1.23) | .09 | 0.27 (0.06, 1.25) | .10 |
|  | 3 | 7.58 (0.90, 63.7) | .06 | 4.46 (0.52, 38.32) | .17 |
| SEIFA-IRSD |  | 0.89 (0.82, 0.97) | .01 | 0.91 (0.83, 0.99) | .03 |
| Location of practice | MMM 2-3 | 1.04 (0.45, 2.42) | .93 | 1.27 (0.54, 3.03) | .58 |
|  | MMM 4-7 | 0.96 (0.39, 2.38) | .94 | 0.92 (0.36, 2.37) | .86 |
| Number of GPs in practice | 2-4 GPs | 1.00 (0.45, 2.20) | .99 | 1.18 (0.50, 2.75) | .71 |
|  | 5-9 GPs | 0.93 (0.38, 2.25) | .87 | 0.96 (0.38, 2.41) | .93 |
|  | >10 GPs | 0.78 (0.30, 2.03) | .61 | 0.87 (0.32, 2.40) | .79 |
| Number of cases |  | 1.03 (0.86, 1.24) | .71 | 1.05 (0.87, 1.27) | .58 |
| Registrar able to reflect on performance | 3 or more cases | 0.48 (0.15, 1.54) | .22 | 0.34 (0.11, 1.03) | .06 |
| Registrar provided with meaningful feedback | 3 or more cases | 2.45 (0.80, 7.52) | .12 | 5.13 (1.58, 16.65) | .006 |
| CT visitor gender | Female | 1.14 (0.69, 1.88) | .60 | 1.18 (0.69, 2.00) | .55 |
| CT visitor Age |  | 0.99 (0.95, 1.03) | .48 | 0.99 (0.94, 1.03) | .53 |
| CT visitor AMG/IMG | IMG | 2.02 (1.03, 3.97) | .04 | 1.76 (0.89, 3.47) | .10 |
| CT Visitor weekly hours |  | 1.03 (1.00, 1.06) | .05 | 1.02 (0.99, 1.05) | .18 |
| CT Visitor years since gaining fellowship |  | 1.01 (0.97, 1.05) | .52 | 1.02 (0.98, 1.07) | .31 |
| CT Visitor years of experience |  | 1.02 (0.96, 1.07) | .55 | 1.00 (0.94, 1.06) | .98 |
| Rurality of Visitor’s practice | RA 2 | 0.80 (0.42, 1.51) | .50 | 0.79 (0.41, 1.53) | .49 |
|  | RA 3-5 | 0.37 (0.19, 0.72) | .003 | 0.40 (0.20, 0.80) | .009 |
| CT Visitor experience as a medical educator | Currently | 1.16 (0.65, 2.04) | .62 | 1.09 (0.59, 2.02) | .79 |
|  | Previously | 0.75 (0.40, 1.40) | .37 | 0.65 (0.34, 1.26) | .20 |
| CT Visitor experience as a GP supervisor | Currently | 1.06 (0.58, 1.93) | .85 | 1.04 (0.56, 1.93) | .90 |
|  | Previously | 2.28 (1.19, 4.34) | .013 | 2.07 (1.05, 4.09) | .04 |
| CT Visitor experience as ACRRM/RACGP examiner | Currently | 0.96 (0.60, 1.54) | .86 | 1.09 (0.63, 1.86) | .77 |
|  | Previously | 0.85 (0.42, 1.75) | .67 | 1.08 (0.49, 2.39) | .85 |
| Consistency of feedback received | Rated 5 | 2.28 (1.44, 3.60) | <.001 | 2.14 (1.34, 3.43) | .002 |
| Quality of feedback received | Rated 5 | 3.31 (1.94, 5.64) | <.001 | 3.42 (1.93, 6.04) | <.001 |
| Discussed patient agenda | Yes | 1.02 (0.63, 1.66) | .92 | 0.94 (0.55, 1.58) | .81 |
| Discussed organisation and flow | Yes | 0.92 (0.57, 1.48) | .73 | 1.06 (0.63, 1.76) | .84 |
| Discussed developing rapport | Yes | 1.03 (0.64, 1.66) | .92 | 0.97 (0.59, 1.59) | .91 |
| Discussed contextual factors | Yes | 1.28 (0.75, 2.19) | .36 | 1.17 (0.66, 2.08) | .59 |
| Discussed time management | Yes | 1.50 (0.98, 2.30) | .06 | 1.32 (0.81, 2.16) | .26 |
| Discussed management planning | Yes | 1.14 (0.73, 1.80) | .56 | 1.27 (0.79, 2.04) | .32 |
| Discussed appropriate medications | Yes | 1.16 (0.71, 1.91) | .54 | 1.09 (0.64, 1.84) | .75 |
| Discussed appropriate investigations | Yes | 1.46 (0.96, 2.22) | .08 | 1.72 (1.10, 2.68) | .02 |
| Discussed patient follow-up | Yes | 0.60 (0.37, 0.98) | .04 | 0.53 (0.31, 0.89) | .02 |
| Discussed dealing with uncertainty | Yes | 1.76 (1.12, 2.77) | .01 | 1.71 (1.05, 2.76) | .03 |
| Discussed physical examination | Yes | 0.97 (0.62, 1.53) | .91 | 0.90 (0.56, 1.44) | .67 |

# Supplementary Table 9. Comparison of Full and Multiple Imputation (MI) multivariable models including all covariates of interest for CT visitor educational usefulness rating.

|  |  | **Full models** | | **MI models** | |
| --- | --- | --- | --- | --- | --- |
| **Covariate** | **Class** | **OR (95% CI)** | **p-value** | **OR (95% CI)** | **p-value** |
| Type of assessment | Video/phone | 0.28 (0.12, 0.69) | .006 | 0.33 (0.13, 0.84) | .02 |
|  | CNA CTV | 0.45 (0.13, 1.55) | .21 | 0.46 (0.13, 1.69) | .24 |
|  | CBD CTV | 0.94 (0.16, 5.49) | .94 | 0.98 (0.17, 5.64) | .98 |
| Registrar gender | Female | 0.99 (0.61, 1.60) | .96 | 0.88 (0.54, 1.45) | .62 |
| Registrar Age |  | 0.99 (0.95, 1.04) | .78 | 0.98 (0.94, 1.03) | .47 |
| Registrar AMG/IMG | IMG | 1.37 (0.73, 2.57) | .33 | 1.39 (0.70, 2.77) | .34 |
| Registrar weekly hours |  | 1.00 (0.92, 1.08) | .95 | 0.98 (0.90, 1.07) | .60 |
| Registrar training term | GPT 2 | 0.76 (0.42, 1.36) | .36 | 0.71 (0.38, 1.31) | .27 |
|  | GPT 3 | 0.52 (0.24, 1.10) | .09 | 0.56 (0.26, 1.20) | .14 |
| Registrar FT/PT | Part time | 1.31 (0.32, 5.30) | .71 | 0.91 (0.2, 4.09) | .90 |
| Registrar pathway | Rural | 0.64 (0.21, 1.94) | .44 | 0.62 (0.20, 1.95) | .42 |
| RTO | 2 | 0.97 (0.24, 3.96) | .97 | 0.60 (0.13, 2.73) | .51 |
|  | 3 | 1.10 (0.15, 7.81) | .93 | 1.09 (0.14, 8.33) | .93 |
| SEIFA-IRSD |  | 1.08 (0.98, 1.19) | .11 | 1.06 (0.96, 1.18) | .26 |
| Location of practice | MMM 2-3 | 1.08 (0.35, 3.30) | .89 | 1.00 (0.33, 3.02) | .99 |
|  | MMM 4-7 | 1.75 (0.49, 6.19) | .39 | 1.42 (0.40, 5.03) | .59 |
| Number of GPs in practice | 2-4 GPs | 1.19 (0.42, 3.38) | .75 | 1.17 (0.45, 3.07) | .75 |
|  | 5-9 GPs | 0.96 (0.34, 2.74) | .94 | 1.12 (0.41, 3.05) | .83 |
|  | >10 GPs | 1.00 (0.27, 3.67) | .99 | 1.09 (0.31, 3.88) | .89 |
| Number of cases |  | 0.88 (0.69, 1.13) | .32 | 0.84 (0.65, 1.09) | .19 |
| Registrar able to reflect on performance | 3 or more cases | 2.31 (0.92, 5.78) | .07 | 2.15 (0.86, 5.38) | .10 |
| Registrar provided with meaningful feedback | 3 or more cases | 0.51 (0.16, 1.56) | .24 | 0.61 (0.18, 2.00) | .41 |
| CT visitor gender | Female | 0.64 (0.30, 1.36) | .25 | 0.60 (0.29, 1.27) | .19 |
| CT visitor Age |  | 1.00 (0.94, 1.05) | .89 | 0.99 (0.94, 1.04) | .73 |
| CT visitor AMG/IMG | IMG | 0.74 (0.30, 1.80) | .50 | 0.63 (0.24, 1.65) | .35 |
| CT Visitor weekly hours |  | 1.03 (1.00, 1.07) | .07 | 1.03 (0.99, 1.07) | .10 |
| CT Visitor years since gaining fellowship |  | 0.99 (0.95, 1.04) | .78 | 0.99 (0.95, 1.04) | .77 |
| CT Visitor years of experience |  | 1.05 (0.97, 1.12) | .21 | 1.05 (0.98, 1.13) | .16 |
| Rurality of Visitor’s practice | RA 2 | 0.50 (0.15, 1.60) | .24 | 0.63 (0.21, 1.88) | .41 |
|  | RA 3-5 | 0.34 (0.13, 0.92) | .03 | 0.41 (0.15, 1.13) | .09 |
| CT Visitor experience as a medical educator | Currently | 1.36 (0.60, 3.08) | .46 | 1.17 (0.53, 2.58) | .70 |
|  | Previously | 0.41 (0.15, 1.14) | .09 | 0.38 (0.14, 1.01) | .05 |
| CT Visitor experience as a GP supervisor | Currently | 1.74 (0.76, 4.01) | .19 | 1.74 (0.76, 3.98) | .19 |
|  | Previously | 6.67 (2.05, 21.7) | .002 | 6.20 (1.99, 19.33) | .002 |
| CT Visitor experience as ACRRM/RACGP examiner | Currently | 0.92 (0.47, 1.80) | .81 | 0.91 (0.46, 1.80) | .78 |
|  | Previously | 0.24 (0.06, 0.97) | .05 | 0.27 (0.07, 0.99) | .05 |
| Discussed patient agenda | Yes | 1.20 (0.69, 2.06) | .52 | 1.22 (0.69, 2.15) | .50 |
| Discussed organisation and flow | Yes | 1.65 (0.99, 2.73) | .05 | 1.46 (0.87, 2.46) | .15 |
| Discussed developing rapport | Yes | 0.64 (0.37, 1.13) | .13 | 0.65 (0.36, 1.17) | .15 |
| Discussed contextual factors | Yes | 0.96 (0.50, 1.85) | .91 | 1.11 (0.57, 2.18) | .76 |
| Discussed time management | Yes | 0.94 (0.52, 1.70) | .83 | 0.87 (0.48, 1.58) | .65 |
| Discussed management planning | Yes | 1.52 (0.87, 2.64) | .14 | 1.52 (0.86, 2.69) | .15 |
| Discussed appropriate medications | Yes | 1.35 (0.77, 2.36) | .30 | 1.26 (0.72, 2.18) | .42 |
| Discussed appropriate investigations | Yes | 1.25 (0.70, 2.22) | .45 | 1.18 (0.64, 2.18) | .59 |
| Discussed patient follow-up | Yes | 1.27 (0.73, 2.23) | .40 | 1.01 (0.56, 1.81) | .98 |
| Discussed dealing with uncertainty | Yes | 0.81 (0.48, 1.36) | .42 | 0.82 (0.48, 1.39) | .45 |
| Discussed physical examination | Yes | 1.21 (0.72, 2.04) | .47 | 1.23 (0.70, 2.16) | .47 |

# Supplementary Table 10. Multivariable sensitivity analysis – Registrar perceived educational usefulness rating with quality of feedback omitted.

|  | | **Univariate** | | **Adjusted** | |
| --- | --- | --- | --- | --- | --- |
| **Covariate** | **Class** | **OR [95% CI]** | **p** | **OR [95% CI]** | **p** |
| Consistency of feedback | Rated 5 | 1.69 (1.26, 2.28) | <.001 | 1.34 (0.97, 1.84) | .08 |
| Registrar AMG/IMG | IMG | 1.39 (0.94, 2.06) | .10 | 1.41 (0.92, 2.15) | .11 |
| Type of Assessment | CBD CTV | 1.89 (0.64, 5.62) | .25 | 1.16 (0.29, 4.68) | .84 |
|  | CNA CTV | 1.93 (1.07, 3.49) | .03 | 1.38 (0.65, 2.91) | .41 |
|  | Video/phone | 1.21 (0.78, 1.86) | .39 | 1.18 (0.65, 2.14) | .59 |
| Registrar provided with meaningful feedback | 3 or more cases | 6.25 (3.26, 12.0) | <.001 | 5.06 (2.56, 10.0) | <.001 |
| RTO | 2 | 0.26 (0.10, 0.68) | .006 | 0.37 (0.15, 0.95) | .04 |
|  | 3 | 2.17 (0.87, 5.42) | .10 | 4.32 (1.11, 16.9) | .04 |
| Discussed consideration of patient’s agenda | Yes | 1.43 (1.06, 1.92) | .02 | 1.00 (0.72, 1.41) | .98 |
| Discussed organisation and flow | Yes | 1.30 (0.96, 1.76) | .09 | 1.06 (0.74, 1.51) | .75 |
| Discussed developing rapport | Yes | 1.36 (1.04, 1.79) | .03 | 0.99 (0.70, 1.40) | .97 |
| Discussed specific contextual factors | Yes | 2.30 (1.63, 3.25) | <.001 | 1.56 (1.03, 2.35) | .04 |
| Discussed management planning | Yes | 1.94 (1.45, 2.58) | <.001 | 1.34 (0.97, 1.85) | .08 |
| Discussed appropriate medications | Yes | 1.69 (1.22, 2.34) | .002 | 1.09 (0.74, 1.63) | .66 |
| Discussed appropriate investigations | Yes | 1.49 (1.11, 2.00) | .007 | 1.15 (0.81, 1.64) | .42 |
| Discussed patient follow-up | Yes | 1.71 (1.26, 2.33) | <.001 | 1.17 (0.81, 1.67) | .40 |
| Discussed dealing with uncertainty | Yes | 1.90 (1.41, 2.56) | <.001 | 1.45 (1.02, 2.06) | .04 |
| Discussed physical examination | Yes | 1.52 (1.14, 2.02) | .004 | 1.08 (0.77, 1.51) | .66 |
| Number of cases |  | 0.92 (0.83, 1.02) | .10 | 0.93 (0.82, 1.05) | .22 |

# Supplementary Table 11. Multivariable sensitivity analysis – Registrar perceived likelihood to change practice with quality of feedback omitted.

|  | | **Univariate** | | **Adjusted** | | |
| --- | --- | --- | --- | --- | --- | --- |
| **Covariate** | **Class** | **OR [95% CI]** | **p** | | **OR [95% CI]** | **p** |
| Consistency of feedback | Rated 5 | 2.09 (1.57, 2.79) | <.001 | | 2.00 (1.36, 2.95) | <.001 |
| CT Visitor experience as ACRRM/RACGP examiner | Currently | 1.34 (0.95, 1.89) | .09 | | 1.30 (0.90, 1.89) | .16 |
|  | Previously | 1.43 (0.86, 2.36) | .16 | | 1.92 (1.03, 3.55) | .04 |
| Registrar AMG/IMG | IMG | 1.85 (1.26, 2.71) | .002 | | 1.80 (1.09, 2.96) | .02 |
| CT Visitor gender | Female | 1.33 (0.92, 1.92) | .13 | | 1.37 (0.89, 2.09) | .15 |
| Type of Assessment | CBD CTV | 1.18 (0.39, 3.50) | .77 | | 0.67 (0.08, 5.45) | .71 |
|  | CNA CTV | 2.56 (1.44, 4.55) | .001 | | 1.34 (0.50, 3.63) | .56 |
|  | Video/phone | 1.30 (0.83, 2.05) | .26 | | 1.07 (0.47, 2.43) | .88 |
| Registrar able to reflect on performance | 3 or more cases | 2.03 (1.20, 3.44) | .009 | | 0.92 (0.40, 2.10) | .84 |
| Registrar provided with meaningful feedback | 3 or more cases | 3.42 (1.88, 6.24) | <.001 | | 3.78 (1.52, 9.41) | .004 |
| RTO | 2 | 0.31 (0.12, 0.80) | .02 | | 0.38 (0.13, 1.07) | .07 |
|  | 3 | 1.07 (0.49, 2.33) | .87 | | 1.07 (0.20, 5.83) | .94 |
| Discussed consideration of patient’s agenda | Yes | 1.50 (1.11, 2.04) | .009 | | 1.17 (0.75, 1.84) | .49 |
| Discussed organisation and flow | Yes | 1.33 (1.01, 1.75) | .04 | | 1.01 (0.66, 1.53) | .98 |
| Discussed developing rapport | Yes | 1.22 (0.93, 1.61) | .15 | | 0.91 (0.60, 1.37) | .65 |
| Discussed specific contextual factors | Yes | 1.42 (1.03, 1.97) | .03 | | 1.06 (0.62, 1.80) | .84 |
| Discussed time management | Yes | 1.46 (1.12, 1.92) | .006 | | 1.69 (1.13, 2.54) | .01 |
| Discussed management planning | Yes | 1.68 (1.27, 2.22) | <.001 | | 1.20 (0.82, 1.77) | .36 |
| Discussed appropriate medications | Yes | 1.67 (1.25, 2.24) | <.001 | | 1.21 (0.76, 1.91) | .42 |
| Discussed appropriate investigations | Yes | 1.62 (1.22, 2.17) | .001 | | 1.59 (1.05, 2.41) | .03 |
| Discussed patient follow-up | Yes | 1.31 (0.98, 1.76) | .07 | | 0.99 (0.65, 1.49) | .95 |
| Discussed dealing with uncertainty | Yes | 1.35 (1.01, 1.81) | .04 | | 1.11 (0.74, 1.67) | .62 |
| Discussed physical examination | Yes | 1.33 (0.99, 1.78) | .06 | | 1.04 (0.69, 1.59) | .85 |

# Supplementary Table 12. Multivariable sensitivity analysis – Registrar perceived likelihood to change learning/training with quality of feedback omitted.

|  | | **Univariate** | | **Adjusted** | |
| --- | --- | --- | --- | --- | --- |
| **Covariate** | **Class** | **OR [95% CI]** | **p** | **OR [95% CI]** | **p** |
| Registrar term | GPT 2 | 1.10 (0.83, 1.44) | .51 | 0.98 (0.67, 1.43) | .91 |
|  | GPT 3 | 0.72 (0.47, 1.10) | .13 | 0.40 (0.21, 0.76) | .005 |
| Consistency of feedback | Rated 5 | 2.32 (1.72, 3.12) | <.001 | 2.33 (1.55, 3.50) | <.001 |
| Registrar AMG/IMG | IMG | 2.67 (1.83, 3.90) | <.001 | 2.94 (1.67, 5.18) | <.001 |
| CT Visitor AMG/IMG | IMG | 1.39 (0.86, 2.26) | .18 | 1.41 (0.81, 2.46) | .22 |
| Type of Assessment | CBD CTV | 0.89 (0.30, 2.60) | .83 | 0.29 (0.03, 3.23) | .31 |
|  | CNA CTV | 2.09 (1.22, 3.56) | .007 | 1.83 (0.67, 5.01) | .24 |
|  | Video/phone | 1.30 (0.85, 1.99) | .24 | 1.24 (0.55, 2.81) | .60 |
| Registrar provided with meaningful feedback | 3 or more cases | 4.32 (2.13, 8.79) | <.001 | 2.35 (0.93, 5.93) | .07 |
| RTO | 2 | 0.27 (0.09, 0.83) | .02 | 0.36 (0.10, 1.34) | .13 |
|  | 3 | 1.41 (0.69, 2.89) | .35 | 2.37 (0.44, 12.7) | .31 |
| Discussed consideration of patient’s agenda | Yes | 1.32 (0.98, 1.79) | .07 | 0.94 (0.61, 1.45) | .80 |
| Discussed developing rapport | Yes | 1.25 (0.94, 1.65) | .12 | 0.96 (0.62, 1.49) | .86 |
| Discussed specific contextual factors | Yes | 1.79 (1.30, 2.46) | <.001 | 1.15 (0.70, 1.87) | .59 |
| Discussed time management | Yes | 1.38 (1.06, 1.79) | .02 | 1.36 (0.94, 1.97) | .10 |
| Discussed management planning | Yes | 1.81 (1.37, 2.40) | <.001 | 1.43 (0.95, 2.15) | .09 |
| Discussed appropriate medications | Yes | 1.84 (1.40, 2.43) | <.001 | 1.21 (0.77, 1.90) | .40 |
| Discussed appropriate investigations | Yes | 1.76 (1.35, 2.29) | <.001 | 1.51 (1.02, 2.22) | .04 |
| Discussed dealing with uncertainty | Yes | 1.62 (1.22, 2.15) | <.001 | 1.68 (1.10, 2.54) | .02 |
| Discussed physical examination | Yes | 1.48 (1.12, 1.96) | .005 | 0.96 (0.64, 1.45) | .86 |
| SEIFA-IRSD |  | 0.95 (0.90, 1.01) | .09 | 0.95 (0.88, 1.02) | .16 |
| Registrar age |  | 1.03 (1.00, 1.05) | .02 | 1.00 (0.96, 1.04) | .96 |
| CT Visitor weekly hours |  | 1.01 (1.00, 1.03) | .16 | 1.01 (0.99, 1.03) | .15 |

# Supplementary Table 13. Multivariable sensitivity analysis – CT visitor perceived overall educational usefulness rating

|  | | **Univariate** | | **Adjusted** | |
| --- | --- | --- | --- | --- | --- |
| **Covariate** | **Class** | **OR [95% CI]** | **p** | **OR [95% CI]** | **p** |
| Rurality of Visitors practice | Inner regional | 0.57 (0.32, 1.00) | .05 | 0.68 (0.36, 1.28) | .23 |
|  | Outer regional | 0.77 (0.45, 1.32) | .34 | 0.64 (0.33, 1.22) | .17 |
| CT Visitor experience as medical educator | Currently | 0.78 (0.49, 1.24) | .29 | 0.89 (0.52, 1.53) | .68 |
|  | Previously | 0.50 (0.23, 1.08) | .08 | 0.62 (0.28, 1.35) | .23 |
| CT Visitor experience as GP Supervisor | Currently | 1.34 (0.83, 2.17) | .24 | 1.22 (0.72, 2.07) | .47 |
|  | Previously | 2.12 (1.12, 4.00) | .02 | 2.55 (1.27, 5.13) | .009 |
| CT Visitor experience as ACRRM/RACGP examiner | Currently | 1.43 (0.90, 2.28) | .13 | 1.49 (0.89, 2.48) | .13 |
|  | Previously | 0.78 (0.35, 1.75) | .55 | 0.59 (0.28, 1.27) | .18 |
| CT Visitor gender | Female | 0.60 (0.37, 0.98) | .04 | 0.51 (0.29, 0.91) | .02 |
| Type of Assessment | CBD CTV | 0.66 (0.19, 2.32) | .52 | 0.61 (0.17, 2.22) | .46 |
|  | CNA CTV | 1.02 (0.51, 2.03) | .95 | 0.97 (0.42, 2.22) | .94 |
|  | Video/phone | 0.63 (0.39, 1.03) | .07 | 0.55 (0.29, 1.04) | .07 |
| Registrar able to reflect on performance | 3 or more cases | 2.07 (1.32, 3.25) | .002 | 1.60 (0.97, 2.65) | .07 |
| Registrar provided with meaningful feedback | 3 or more cases | 1.92 (1.08, 3.41) | .03 | 1.16 (0.57, 2.37) | .68 |
| RTO | 2 | 0.53 (0.24, 1.20) | .13 | 0.70 (0.27, 1.79) | .46 |
|  | 3 | 1.83 (0.78, 4.26) | .16 | 1.07 (0.31, 3.69) | .91 |
| Discussed consideration of patient’s agenda | Yes | 1.40 (1.01, 1.93) | .04 | 1.31 (0.90, 1.92) | .16 |
| Discussed organisation and flow | Yes | 1.57 (1.15, 2.15) | .004 | 1.53 (1.07, 2.18) | .02 |
| Discussed management planning | Yes | 1.52 (1.15, 2.02) | .003 | 1.39 (0.97, 1.99) | .07 |
| Discussed appropriate medications | Yes | 1.36 (0.97, 1.93) | .08 | 1.12 (0.75, 1.67) | .58 |
| Discussed appropriate investigations | Yes | 1.33 (0.99, 1.77) | .06 | 1.22 (0.86, 1.72) | .26 |
| Discussed patient follow-up | Yes | 1.37 (0.98, 1.93) | .07 | 0.94 (0.64, 1.40) | .78 |
| Discussed physical examination | Yes | 1.41 (1.07, 1.86) | .02 | 1.30 (0.95, 1.78) | .10 |
| SEIFA-IRSD |  | 1.04 (0.99, 1.10) | .11 | 1.06 (1.00, 1.12) | .06 |
